# Supplementary material for: Metformin Retards Aging in C. elegans by Altering Microbial Folate and Methionine Metabolism
Source: Cell. 2013 Mar 28;153(1):228–39. doi: 10.1016/j.cell.2013.02.035 (PMC3898468; doi:10.1016/j.cell.2013.02.035)
Supplement: Document S1. Article plus Supplemental Information [file mmc9.pdf]

Cell

Metformin Retards Aging in *C. elegans* by Altering Microbial Folate and Methionine Metabolism

Filipe Cabreiro,[1] Catherine Au,[1,4] Kit-Yi Leung,[2,4] Nuria Vergara-Irigaray,[1] Helena M. Cochemé,[1] Tahereh Noori,[1] David Weinkove,[3] Eugene Schuster,[1] Nicholas D.E. Greene,[2] and David Gems[1,*]
[1]Institute of Healthy Ageing, and G.E.E., University College London, London WC1E 6BT, UK
[2]Neural Development Unit, Institute of Child Health, University College London, London WC1N 1EH, UK
[3]School of Biological and Biomedical Sciences, Durham University, Durham DH1 3LE, UK
[4]These authors contributed equally to this study
*Correspondence: david.gems@ucl.ac.uk
http://dx.doi.org/10.1016/j.cell.2013.02.035

SUMMARY

The biguanide drug metformin is widely prescribed to treat type 2 diabetes and metabolic syndrome, but its mode of action remains uncertain. Metformin also increases lifespan in *Caenorhabditis elegans* cocultured with *Escherichia coli*. This bacterium exerts complex nutritional and pathogenic effects on its nematode predator/host that impact health and aging. We report that metformin increases lifespan by altering microbial folate and methionine metabolism. Alterations in metformin-induced longevity by mutation of worm methionine synthase (*metr-1*) and *S*-adenosylmethionine synthase (*sams-1*) imply metformin-induced methionine restriction in the host, consistent with action of this drug as a dietary restriction mimetic. Metformin increases or decreases worm lifespan, depending on *E. coli* strain metformin sensitivity and glucose concentration. In mammals, the intestinal microbiome influences host metabolism, including development of metabolic disease. Thus, metformin-induced alteration of microbial metabolism could contribute to therapeutic efficacy—and also to its side effects, which include folate deficiency and gastrointestinal upset.

INTRODUCTION

Metformin is the world's most widely prescribed drug, as an oral antihyperglycemic agent for type 2 diabetes (T2D) and in the treatment of metabolic syndrome. However, the real and potential benefits of metformin therapy go beyond its prescribed usage, including reduced risk of cancer (Dowling et al., 2011) and, in animal models, delayed aging, an effect seen in rodents (Anisimov et al., 2011) and in the nematode *Caenorhabditis elegans* (Onken and Driscoll, 2010). The mechanisms underlying these positive effects remain unclear. One possibility is that metformin recapitulates the effects of dietary restriction (DR), the controlled reduction of food intake that can improve late-life health and increases lifespan in organisms ranging from nematodes and fruit flies to rodents and rhesus monkeys (Mair and Dillin, 2008). In mammals, the action of metformin is partly mediated by AMPK activation, which results in downregulation of TOR and the IGF-1/AKT pathways to reduce energy-consuming processes (Pierotti et al., 2012). An unexplored possibility is that metformin alters mammalian physiology via its effects on gut microbiota (Bytzer et al., 2001).

The gut microbiome (or microbiota) plays a major role in the effects of nutrition on host metabolic status (Nicholson et al., 2012), as well as contributing to metabolic disorders such as obesity, diabetes, metabolic syndrome, autoimmune disorders, inflammatory bowel disease, liver disease, and cancer (Delzenne and Cani, 2011; Kau et al., 2011; Nicholson et al., 2012). It may also influence the aging process (Ottaviani et al., 2011). It has been argued that the host and its symbiotic microbiome acting in association (holobiont) should be considered as a unit of selection in evolution (Zilber-Rosenberg and Rosenberg, 2008). Coevolution of microbiota facilitates host adaptation by enabling e.g., nutrient acquisition, vitamin synthesis, xenobiotic detoxification, immunomodulation, and gastrointestinal maturation. In return, the host provides a sheltered incubator with nutrients (Bäckhed et al., 2005). Thus, the two components of the holobiont are symbiotic, but microbiota can also be commensal or pathogenic.

Defining interactions between drug therapy, microbiome and host physiology is experimentally challenging given the complex and heterogeneous nature of mammalian gut microbiota. Here simple animal models amenable to genetic manipulation can be helpful. For example, in the fruit fly *Drosophila*, microbiota modulates host development and metabolic homeostasis via the TOR pathway (Storelli et al., 2011).

C. elegans is particularly convenient for such studies because under standard culture conditions only a single microbe is present (as a food source): the human gut bacterium *Escherichia coli* (Brenner, 1974). Active bacterial metabolism is a critical nutritional requirement for *C. elegans*, the absence of which retards development and extends lifespan (Lenaerts et al., 2008).

 ©2013 Elsevier Inc.

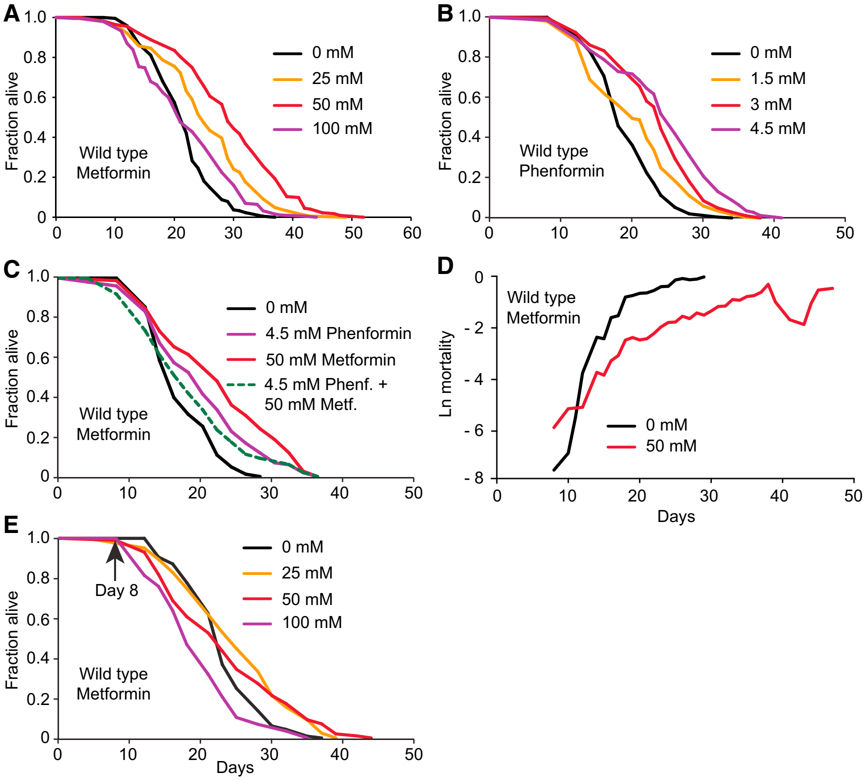

Figure 1. The Biguanide Drugs Phenformin and Metformin Decelerate Aging in *C. elegans*

(A and B) Metformin (A) and phenformin (B) extend lifespan in a dose-dependent manner. Phenformin alters fecundity and reduces body size (see Figures S1A–S1D).

(C) Phenformin (4.5 mM) does not increase lifespan in the presence of 50 mM metformin, consistent with similar mechanism of drug action.

(D) Metformin decreases the exponential increase in age-related mortality (for survival curve see Figure S1E).

(E) Later-life administration (day 8) of metformin increases lifespan at lower concentrations (25, but not 50 or 100 mM).

See also Figure S1. For statistics, see Table S1.

Moreover, worms are sometimes long-lived on mutant *E. coli* with metabolic defects (Saiki et al., 2008; Virk et al., 2012) and on microbial species thought to enhance human health, e.g., from the genera *Lactobacillus* and *Bifidobacterium* (Ikeda et al., 2007). These observations suggest that *E. coli* plays a more active role in *C. elegans* nutrition and metabolism than as a mere food source, and in some respects acts as microbiota (Lenaerts et al., 2008). *C. elegans* has also been used extensively to identify genes that specify endocrine, metabolic, and dietary regulation of aging (Kenyon, 2010).

In this study, we examine the mechanism by which metformin extends lifespan in *C. elegans*. We report that its effects are mediated by the cocultured *E. coli*, where metformin inhibits bacterial folate and methionine metabolism. This, in turn, leads to altered methionine metabolism in the worm, and increased lifespan. These findings reveal how drug action on host-microbiome interactions can impact health and longevity.

RESULTS

Extension of *C. elegans* Lifespan by Metformin Is Mediated by Live *E. coli*

We first verified the effects on worm lifespan of metformin, and also the more potent biguanide drug phenformin. Metformin at 25, 50, and 100 mM increased mean lifespan by 18%, 36%, and 3% (Figure 1A; Table S1 available online). Phenformin at 1.5, 3, and 4.5 mM also increased lifespan, by 5%, 21%, and 26% (Figure 1B; Table S1). As expected, maximal effects on lifespan of these pharmacologically similar drugs were nonadditive (Figure 1C; Table S1). Metformin reduced the exponential age increase in mortality rate (Figure 1D), demonstrating that it slows aging (at least until day 18) rather than reducing risk of death. Metformin also modestly increased mean lifespan when administered from middle age onward, but only at 25 mM (+8%, $p < 0.001$; Figure 1E; Table S1). In most trials, the DNA replication inhibitor FUdR was used to prevent progeny production, but effects of metformin on lifespan are not FUdR-dependent (Figures S1F and S1G; Table S1) (Onken and Driscoll, 2010). These results confirm the robust effects of biguanide drugs on aging in *C. elegans*.

Interventions altering *E. coli* can affect *C. elegans* lifespan (Garigan et al., 2002; Gems and Riddle, 2000; Saiki et al., 2008). To test the possibility that metformin increases worm lifespan by altering the *E. coli*, we assessed its effects in the absence of bacteria (axenic culture). As expected, culture on axenic medium (Lenaerts et al., 2008) and bacterial deprivation (Kaeberlein et al., 2006) caused an increase in worm lifespan, typical of DR. Under these conditions, metformin did not increase worm lifespan, but instead markedly reduced it (Figures 2A, S2A, and S2B; Table S2).

UV-irradiation of *E. coli* impairs bacterial viability and extends worm lifespan without reducing fertility, suggesting a mechanism distinct from DR (Gems and Riddle, 2000). Under these conditions, metformin still shortened lifespan (−16%, $p < 0.001$; Figure 2B; Table S2). Next, we raised *E. coli* in the presence of metformin and then transferred it to drug-free agar plates. Drug pretreatment of *E. coli* robustly extended worm lifespan (+33%, $p < 0.001$; Figure 2C; Table S2). We conclude that the life-extending effect of metformin is mediated by live *E. coli*. Moreover, in the absence of *E. coli*, metformin shortens *C. elegans* lifespan, likely reflecting drug toxicity.

One possibility is that metformin extends worm lifespan by reducing *E. coli* pathogenicity. Proliferating *E. coli* block the alimentary canal in older worms, and antibiotic treatment can both prevent this proliferation and increase worm lifespan

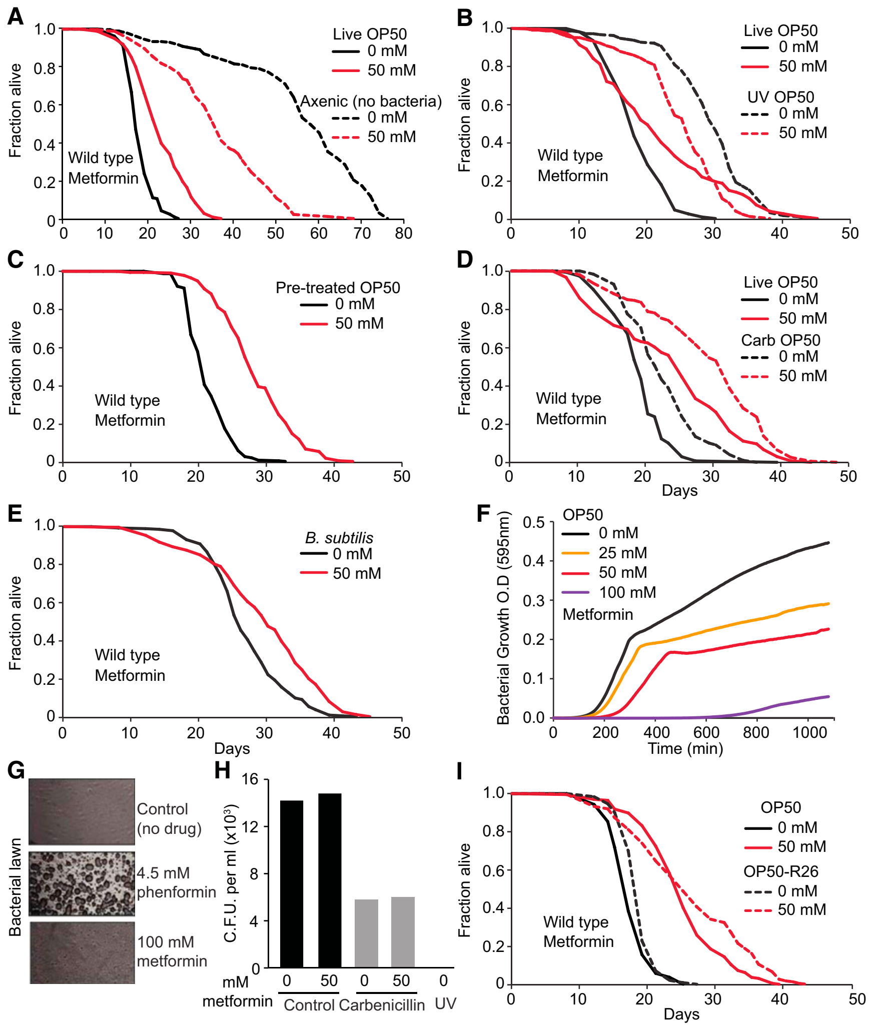

Figure 2. Metformin Extending Effects on *C. elegans* Lifespan Require Live Bacteria

(A) Metformin shortens lifespan of *C. elegans* cultured axenically (i.e., in the absence of *E. coli*).

(B) Metformin shortens lifespan of *C. elegans* cultured on UV-irradiated *E. coli* (OP50).

(C) Metformin pretreatment of bacteria is sufficient to extend lifespan.

(D) Metformin extends lifespan in the absence of *E. coli* proliferation (blocked by carbenicillin).

(E) Metformin extends lifespan in the presence of the less pathogenic bacterium *Bacillus subtilis*.

(F) Retardation of bacterial growth by metformin, monitored over an 18 hr period.

(G) Biguanide drugs cause altered bacterial lawn morphology.

(H) Bacterial viability is reduced by carbenicillin and UV treatment, but not metformin.

(I) Metformin extends lifespan in the presence of multi-antibiotic resistant *E. coli* OP50-R26.

See also Figure S2. For statistics, see Table S2.

(Garigan et al., 2002). To determine whether metformin extends worm lifespan by preventing *E. coli* proliferation, we tested its effects in the presence of carbenicillin. This antibiotic is bacteriostatic, blocking bacterial proliferation without greatly reducing its viability. Metformin increased lifespan to a similar degree in the absence (+25%) or presence (+24%) of carbenicillin ($p < 0.001$; Figure 2D; Table S2). Thus, metformin does not increase lifespan by preventing bacterial proliferation. Culture of *C. elegans* with *Bacillus subtilis* increases lifespan (Garsin et al., 2003), suggesting that this microbe is less pathogenic to *C. elegans* than *E. coli*. Metformin increased lifespan of worms cultured on *B. subtilis* (+9%, $p < 0.001$; Figure 2E; Table S2). These findings suggest that reduced bacterial pathogenicity is not the cause of metformin-induced longevity.

Biguanides Have Bacteriostatic Effects at Concentrations that Increase Lifespan

Biguanides induced a dose-dependent inhibition of *E. coli* proliferation (Figures 2F and S2C) and an alteration in bacterial lawn morphology (Figure 2G). Similar results were obtained with *B. subtilis* (Figures S2D–S2F). Thus, metformin can also act as an antibiotic. Notably, the drug concentration thresholds for bacterial and worm lifespan effects were similar, and also pH-dependent (Figures S2G and S2H and Table S2).

We then asked if the antibiotic effects of metformin were bacteriocidal or bacteriostatic. When subcultured from metformin plates, *E. coli* showed no reduction in colony forming units (Figure 2H), implying that metformin has bacteriostatic rather than bacteriocidal effects. To probe whether metformin acts via one of the major, known antibiotic mechanisms, we employed the R26 P-group plasmid that confers resistance to carbenicillin, neomycin, kanamycin, tetracycline, streptomycin, gentamicin, mercuric ions, and sulfonamides. However, metformin still extended lifespan in worms on R26-transformed *E. coli* (39%, $p < 0.001$; Figure 2I and Table S2).

What is the property of *E. coli* whose alteration by metformin increases worm lifespan? Coenzyme Q (ubiquinone) deficiency in *E. coli* increases *C. elegans* lifespan due to impairment of bacterial respiration (Saiki et al., 2008). We therefore tested whether metformin can increase lifespan of worms on Q-deficient *ubiG* mutant *E. coli* and found that it does (+20%, $p < 0.001$; Figure 3A). We then tested whether metformin reduces respiration rate in *E. coli* OP50. Although metformin transiently reduced respiration rate, long-term exposure increased it (Figure 3B). Taken together, these findings suggest that metformin's effect on worm lifespan is not caused by inhibition of bacterial respiration.

Lipopolysaccharides (LPS) are the major component of the outer wall of Gram-negative bacteria. The structure of *E. coli* LPS can affect *C. elegans* lifespan (Maier et al., 2010). To test whether metformin action is dependent upon *E. coli* LPS type, we looked at worm lifespan on seven *E. coli* strains with a variety

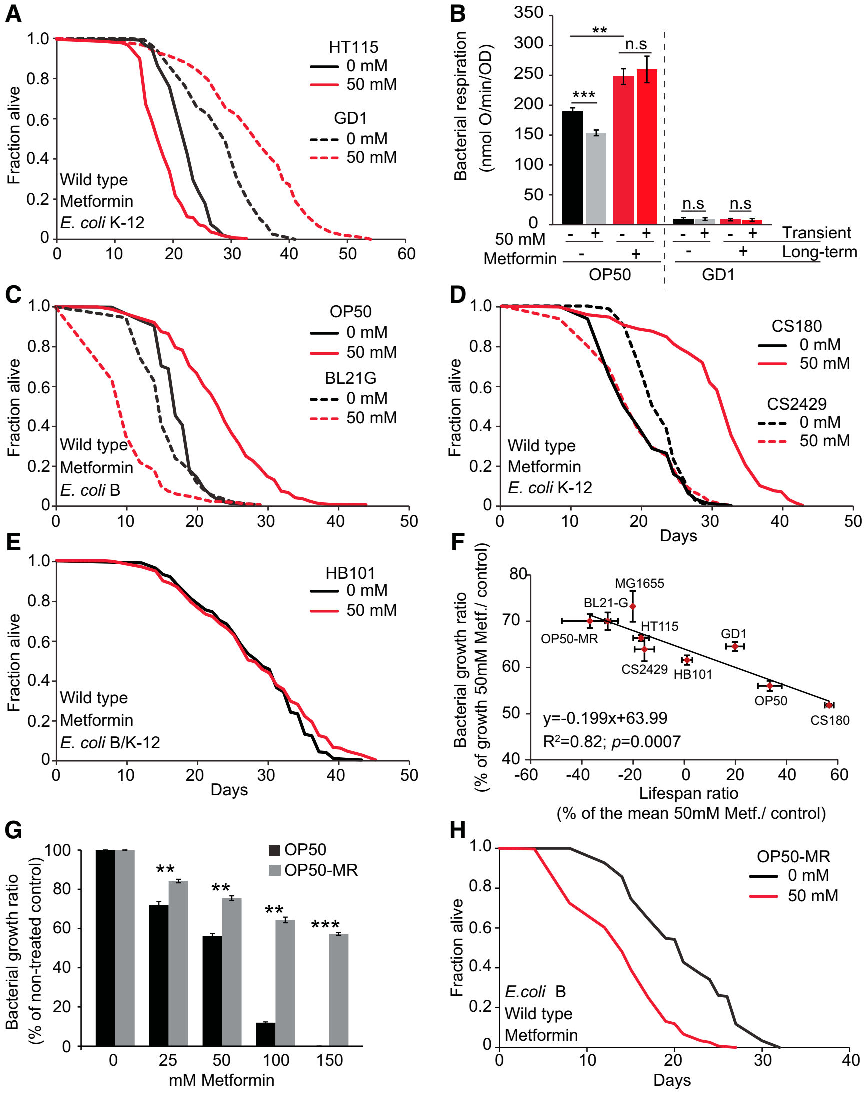

Figure 3. Metformin Effects on *C. elegans* Lifespan Correlate with Effects of Metformin on Bacterial Growth

(A) Metformin extends lifespan in the presence of respiratory-deficient *E. coli* strain GD1.

(B) Growth in the presence of metformin does not impair respiration in *E. coli* OP50.

(C–E) Effects on lifespan are independent of bacterial subgroup (B or K-12) and lipopolysaccharide (LPS) structure. K-12 strains possess longer LPS structures than B strains and CS2429. CS2429 is an LPS truncated mutant derived from isogenic parent strain CS180. HB101 is a B/K-12 hybrid.

(F) Relationship between bacterial growth inhibition by metformin (50 mM) and effects on lifespan among different *E. coli* strains.

(G) OP50-MR *E. coli* is resistant to growth inhibition by metformin. This strain also shows cross-resistance to phenformin (Figures S3C–S3E).

(H) Metformin shortens lifespan in the presence of OP50-MR.

Error bars represent SEM. $^{*}p < 0.05$; $^{**}p < 0.01$; $^{***}p < 0.001$. See also Figure S3. For statistics, see Table S3.

of LPS structures. Although effects of metformin on worm lifespan differed between *E. coli* strains (Figures 3A–3E and S3A and Table S3), this variation did not correlate with the *E. coli* LPS type.

Interestingly, among *E. coli* strains there was a strong positive correlation between the capacity of metformin to increase worm lifespan and to inhibit bacterial growth ($R^2 = 0.82$, $p < 0.0007$; Figure 3F). There was no correlation between bacterial metformin sensitivity and effect on worm lifespan in the absence of metformin ($R^2 = 9.6 \times 10^{-5}$, $p = 0.98$; Figure S3B). This suggests that the capacity of the drug to extend worm lifespan is a function of the microbial sensitivity to growth inhibition by metformin. To test this directly, we isolated a metformin-resistant OP50 derivative (OP50-MR) (Figures 3G and S3C–S3E) that proved to contain eight mutations (see Extended Experimental Procedures). As predicted, on this strain 50 mM metformin shortened worm lifespan (−37%, $p < 0.001$; Figure 3H). We conclude that in metformin-resistant *E. coli* strains, life-shortening toxic effects predominate. However, inhibition of bacterial proliferation per se is not the cause of worm life extension, as already shown (Figure 2D; Table S2).

Metformin Disrupts Folate Metabolism in *E. coli*

It was recently discovered that *C. elegans* live longer on an *E. coli* mutant with reduced folate levels (*aroD*) (Virk et al., 2012). Moreover, metformin can decrease folate levels in patients (Sahin et al., 2007). We therefore asked whether metformin increases worm lifespan by altering bacterial folate metabolism.

Folates are B-group vitamins whose structure incorporates a pteridine ring, *p*-aminobenzoic acid (*p*ABA), and glutamic acid(s). Folates are typically present as the reduced forms, dihydrofolate (DHF) and tetrahydrofolate (THF). THF can be substituted with a variety of one-carbon units (including formyl and methyl groups) that function as a coenzyme in metabolic reactions involving transfer of one-carbon moieties (Figure 4A). These are involved in the biosynthesis of purines and pyrimidines, in amino acid interconversions, and for the provision of methyl groups in methylation reactions (Kwon et al., 2008).

Metformin markedly changed the folate composition in OP50 (Figure 4B), as detected by LC-MS/MS. It increased levels of 5-methyl-THF (+116%, $p = 2.5 \times 10^{-6}$), 5,10-methylene-THF (+99%, $p = 5.9 \times 10^{-6}$), and DHF (+38%, $p = 7.1 \times 10^{-4}$), whereas levels of the remaining folates were decreased. It also increased folate polyglutamylation, particularly n = 6 and 7 glutamates (Figures 4C, S4A, and S4B; Table S4). Folate

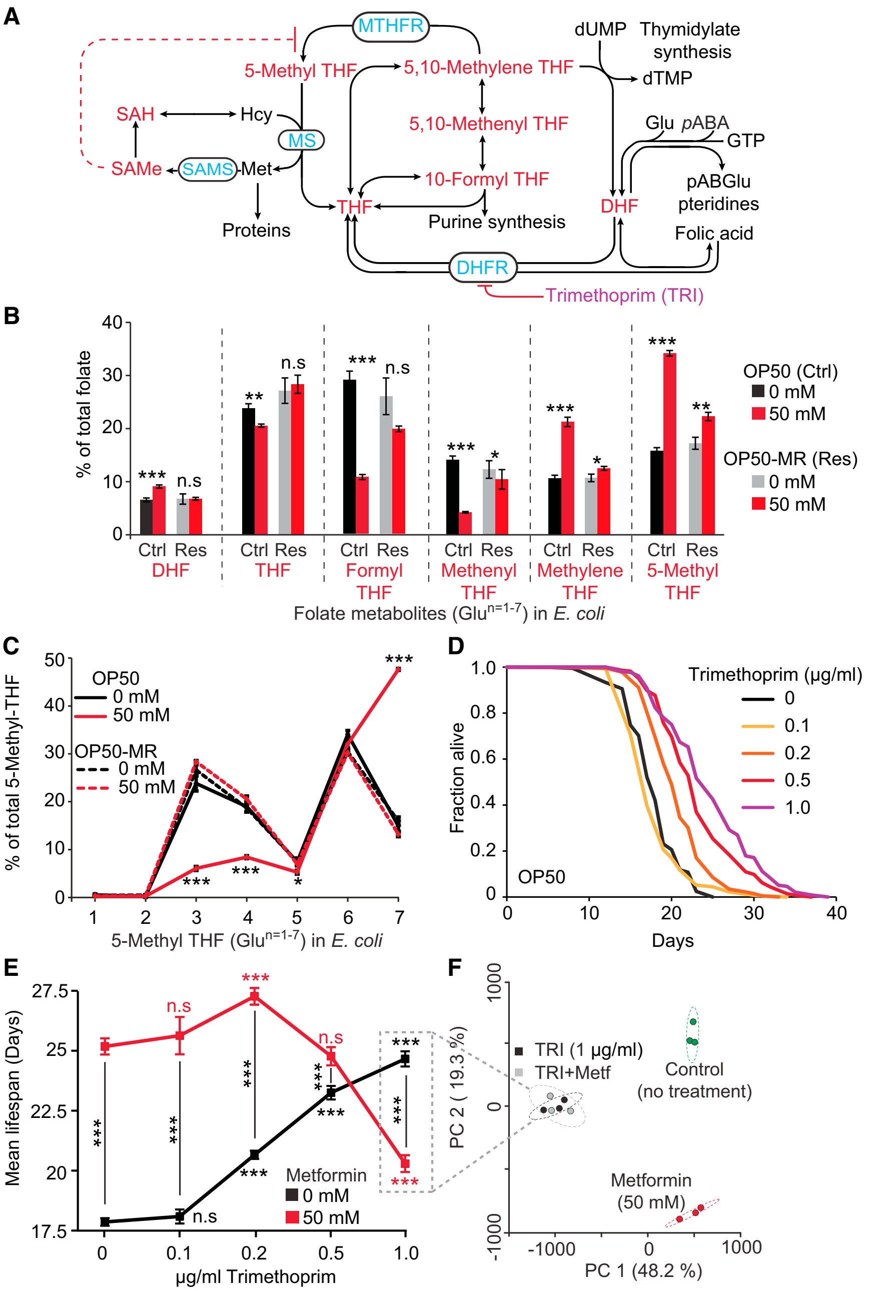

Figure 4. Metformin Inhibits Bacterial Folate Metabolism

(A) The folate and methionine cycles. Metabolites analyzed, red; enzymes, blue; supplements, purple. DHF, dihydrofolate; DHFR, dihydrofolate reductase; Glu, glutamate; Hcy, homocysteine; Met, methionine; MS, methionine synthetase; MTHFR, methylenetetrahydrofolate reductase; *p*ABA, *p*-aminobenzoic acid; SAH, *S*-adenosylhomocysteine; SAMe, *S*-adenosylmethionine; SAMS, *S*-adenosylmethionine synthase; THF, tetrahydrofolate; TRI, trimethoprim. Dotted lines represent feedback loops.

(B) Metformin alters folate homeostasis in *E. coli* OP50 but not OP50-MR. The values for each metabolite are the sum of the values for the different glutamate side chains (1–7) divided by sum of all folate metabolites measured.

(C) Metformin alters 5-methyl-THF polyglutamylation in OP50 but not OP50-MR.

(D) The DHFR inhibitor TRI increases *C. elegans* lifespan in a dose-dependent manner. See Figure S4D for *E. coli* growth retardation by TRI.

(E) Effects of metformin and TRI on lifespan are nonadditive, consistent with similar modes of action.

(F) Principal component analysis (Metaboanalyst) of OP50 metabolites with TRI and metformin. Note that TRI abolishes effects of metformin.

Error bars represent SEM. *$p < 0.05$; **$p < 0.01$; ***$p < 0.001$. See also Figure S4. For statistics, see Table S4.

polyglutamylation increases their retention in the cell, and bioavailability for reactions involving folate-dependent enzymes (Kwon et al., 2008). By contrast, in the resistant strain OP50-MR, metformin did not affect polyglutamylation (Figures 4C and S4C), or DHF levels. 5-Methyl-THF and 5,10-methylene-THF were still increased (Figure 4B), but by only 29% ($p = 0.018$) and 17% ($p = 0.003$). Genome sequencing of OP50-MR revealed a mutation in glyA, which encodes a folate cycle enzyme.

To explore whether metformin effects on bacterial folate metabolism affect worm lifespan, we used the antibiotic trimethoprim (TRI) that inhibits dihydrofolate reductase (DHFR). TRI (0.2, 0.5, and 1 µg/ml) increased lifespan by 16%, 30%, and 38%, respectively ($p < 0.001$) (Figure 4D). By contrast, in the presence of 50 mM metformin, 0.2 µg/ml TRI caused only a slight increase in lifespan (+8%, $p < 0.001$), whereas at higher concentrations it either had no effect (0.5 µg/ml TRI, −2%, $p = 0.17$) or reduced lifespan (1 µg/ml TRI, −19%, $p < 0.001$; Figure 4E). Such nonadditivity was recapitulated in the lack of effect of metformin on metabolic profiles of OP50 when cotreated with 1 µg/ml TRI (Figures 4F and S4E). These nonadditive effects of metformin and TRI imply a shared mechanism of action, suggesting that altered bacterial folate metabolism by metformin increases worm lifespan.

Metformin Disrupts *C. elegans* Methionine Metabolism

To explore whether metformin-induced alterations in microbial folate metabolism increase host lifespan by altering worm folate metabolism, we first examined worm folate profiles under standard culture conditions (agar plates with *E. coli* OP50). In worms, as in humans, 5-methyl-THF was the predominant folate (59%) and treatment with metformin did not alter the ratio of different folate forms (Figure 5A). However, it did decrease

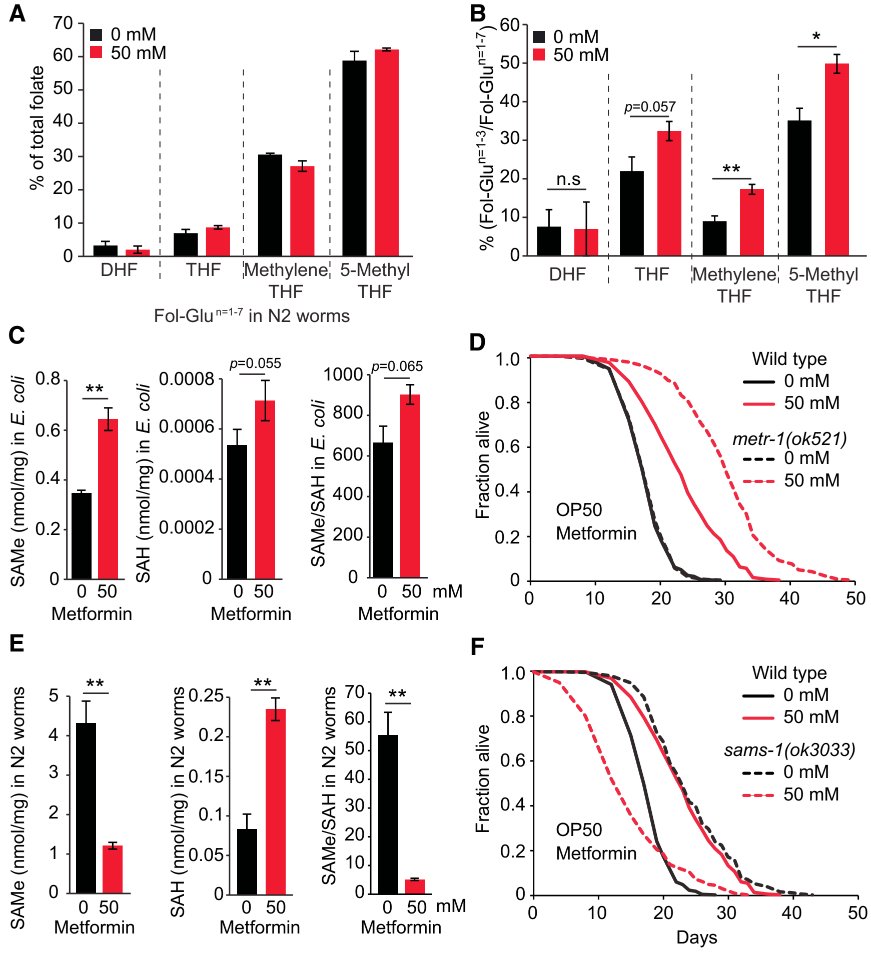

Figure 5. Effect of Metformin on the Methionine Cycle but Not the Folate Cycle in *C elegans*

(A) Effect of metformin on *C. elegans*/*E. coli* system: little effect on nematode folate homeostasis.

(B) Metformin induces a shift toward shorter-chain (n = 1–3) glutamate folate forms in *C. elegans*.

(C) Metformin increases *S*-adenosylmethionine (SAMe) levels in *E. coli* (OP50).

(D) Mutation of *metr-1(ok521)* (methionine synthetase, MS) increases lifespan only in the presence of metformin.

(E) In *C. elegans*, metformin greatly reduces SAMe levels and increases *S*-adenosylhomocysteine (SAH) levels.

(F) Metformin shortens lifespan in *S*-adenosylmethionine synthase-deficient *sams-1 (ok3033)* mutants.

Error bars represent SEM. $^*p < 0.05$; $^{**}p < 0.01$; $^{***}p < 0.001$. See also Figure S5. For statistics, see Table S5.

glutamate chain length (n = 1–3) (Figures 5B and S5; Table S5), suggesting a possible change in the activity of folate-dependent enzymes.

Thus, disruption of microbial folate metabolism increases host lifespan but with little effect on host folate levels. One possibility is that products of other *E. coli* folate-associated pathways influence *C. elegans* lifespan. Inhibition of bacterial methionine synthase (MS) causes 5-methyl-THF accumulation via the "methyl trap" mechanism, so-called because of the irreversible conversion of 5,10-methylene-THF to 5-methyl-THF (Mato et al., 2008) (Figure 4A). Consistent with MS inhibition, metformin not only strongly increased 5-methyl-THF levels but also reduced levels of THF (−14%, p = 0.003) (Figure 4B). Metformin also impaired the bacterial methionine cycle, causing an 86% increase in *S*-adenosylmethionine (SAMe) levels (p = 0.0032) and a 33% increase of *S*-adenosylhomocysteine (SAH) (p = 0.055; Figures 4A and 5C), consistent with the lack of homocysteine (Hcy) remethylation if MS is inhibited. SAMe, the major corepressor of genes encoding enzymes of methionine biosynthesis, also inhibits the folate cycle and reduces methionine production by blocking methylene-THF reductase (MTHFR) (Banerjee and Matthews, 1990) (Figure 4A). Thus, the accumulation of the substrates SAMe, SAH, 5-methyl-THF, and 5,10-methylene-THF, and reduction of the product THF imply that metformin also reduces microbial methionine availability.

This suggests that metformin might increase lifespan by reducing levels of bacterial-derived methionine in the host. To explore this, we employed a *C. elegans* MS mutant, *metr-1(ok521)*, which cannot synthesize methionine and is therefore wholly dependent upon exogenous methionine (Hannich et al., 2009). In the absence of metformin, *metr-1* did not increase worm lifespan (p = 0.85; Figure 5D). Interestingly however, *metr-1* did increase lifespan in the presence of 50 mM metformin (+67%, p < 0.001; Figure 5D). Thus, *metr-1* sensitizes *C. elegans* to the life-extending effects of metformin.

This suggests that microbes are the main source of dietary methionine, but the worms also synthesize some methionine of their own using METR-1. Thus, effects of *metr-1* on lifespan are only detected when dietary methionine levels are reduced. Supporting this scenario, metformin treatment lowered SAMe levels in *C. elegans* (−72%, p = 0.005) and increased SAH levels (+181%, p = 0.002; Figure 5E). In summary, in *E. coli* metformin increases SAMe and 5-methyl-THF. By contrast, in *C. elegans* it decreases SAMe and the SAMe/SAH ratio without affecting 5-methyl-THF levels.

In *C. elegans*, SAMe is synthesized by the SAMe synthase SAMS-1, RNAi knockdown of which extends lifespan (Hansen et al., 2005). Notably, *sams-1* RNAi does not increase *eat-2* mutant lifespan, suggesting a shared mechanism with *eat-2*-induced DR (Ching et al., 2010; Hansen et al., 2005). If metformin increases lifespan by the same mechanism as loss of *sams-1*, then metformin should not increase lifespan in the absence of *sams-1*. To test this, we employed a *sams-1(ok3033)* null mutant that, as expected, extended lifespan (+35%, p < 0.001;

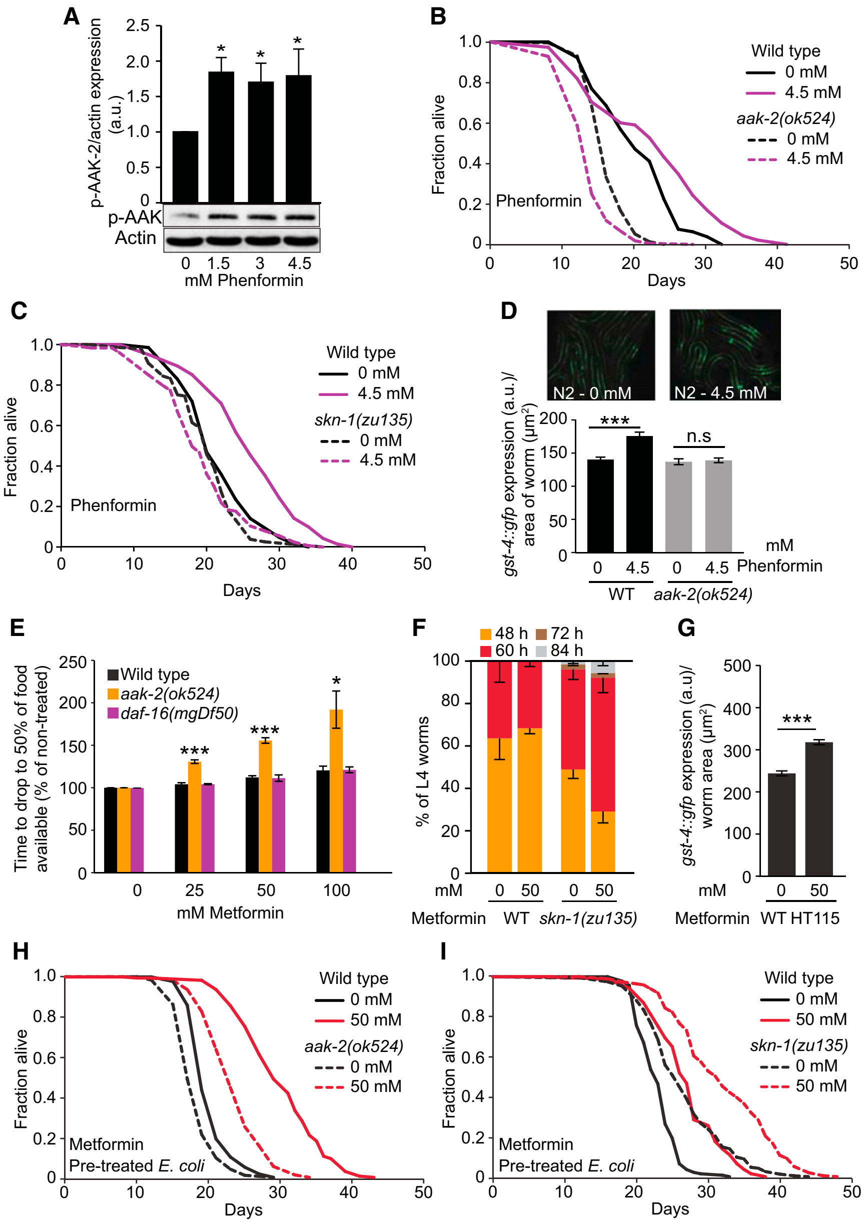

Figure 6. AMP kinase and SKN-1 Protect Against Biguanide Toxicity

(A) Phenformin increases pAAK-2 levels, suggesting AMPK activation (2-day-old adults).

(B) Phenformin shortens lifespan in *aak-2(ok524)* AMPK loss-of-function mutants.

(C) Phenformin does not extend lifespan in *skn-1(zu135)* mutants.

(D) AMPK-dependent induction of expression by phenformin of SKN-1-activated reporter *gst-4::gfp* in L4 animals.

(E) *aak-2(ok524)* but not *daf-16(mgDf50)* mutants are hypersensitive to growth inhibition by metformin, as measured by the food clearance assay.

(F) *skn-1(zu135)* increases sensitivity to growth inhibition by metformin.

(G) Metformin increases expression of *gst-4::gfp* under conditions that do not increase lifespan (maintenance on *E. coli* HT115).

(H) Life extension by metformin pretreatment of *E. coli* is partially AMPK-dependent.

(I) Life extension by metformin pretreatment of *E. coli* is not SKN-1-dependent.

Error bars represent SEM of at least three independent biological replicates. *$p < 0.05$; **$p < 0.01$; ***$p < 0.001$. See also Figure S6. For statistics, see Table S6.

Figure 5F). Strikingly, in a *sams-1* mutant, metformin reduced lifespan (−38%, $p < 0.001$), reminiscent of the effect of metformin on *eat-2* mutants (Onken and Driscoll, 2010). These results suggest the possibility that metformin and *eat-2*-induced DR act by similar disruptions of methionine-associated functions.

AMP Kinase and SKN-1 Protect *C. elegans* Against Metformin Toxicity

Metformin-induced longevity requires the worm AMP-dependent protein kinase (AMPK) (Onken and Driscoll, 2010). This is consistent with the fact that biguanide drugs activate AMPK (Hawley et al., 2003). However, if extension of *C. elegans* lifespan by biguanide drugs is mediated by *E. coli*, why should this effect require the worm AMPK? To explore this, we first tested whether biguanides activate worm AMPK, by measuring phosphorylation of Thr-172 in the worm AMPKα subunit AAK-2. Phenformin, but not metformin, detectably increased pAMPK levels (Figures 6A and S6A), perhaps reflecting the greater membrane permeability of phenformin. We then verified the AMPK-dependence of the effect of biguanides on worm lifespan in the presence of *E. coli*. Lifespan in *aak-2* mutants was not increased by either metformin (Figure S6B; Table S6), as previously noted (Onken and Driscoll, 2010), or phenformin (Figure 6B). In fact, phenformin reduced lifespan (−15%, $p < 0.001$; Table S6). Notably, the metformin-induced deceleration of the age increase in mortality rate was still present in *aak-2* mutants, but initial mortality rates were markedly greater (Figure S6C), consistent with increased sensitivity to metformin toxicity.

The life-extending effects of both biguanides also required the SKN-1 Nrf2 transcription factor (Figures 6C and S6H), and induced expression of the SKN-1 target *gst-4* (glutathione *S*-transferase 4) in an AMPK-dependent fashion (Figures 6D and S6I–S6K), consistent with previous findings (Onken and Driscoll, 2010). Thus, both biguanides cause AMPK-dependent activation of SKN-1, and induce detoxification gene expression.

Our findings imply that the impact of metformin on worm lifespan reflects the sum of indirect, *E. coli*-mediated life-extending effects and direct life-shortening effects. A possible interpretation of the AMPK and SKN-1 dependence of biguanide effects

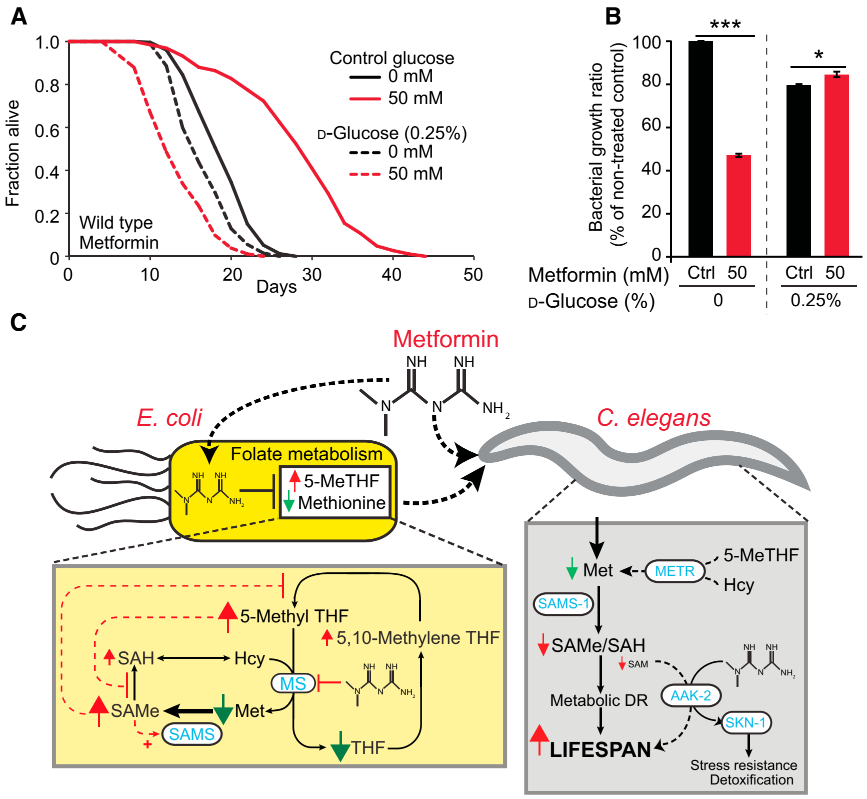

Figure 7. High Glucose Diet Suppresses Metformin-Induced Life Extension

(A) Metformin decreases lifespan on 0.25% D-glucose. See Figure S7A for 1% D-glucose.

(B) Metformin does not inhibit bacterial growth in the presence of 0.25% D-glucose.

(C) Scheme summarizing direct and indirect effects of metformin on the *C. elegans/E. coli* system. Dotted lines indicate hypothetical feedback loops.

Error bars represent SEM of at least three independent biological replicates. $^{*}p < 0.05$; $^{**}p < 0.01$; $^{***}p < 0.001$. See also Figure S7. For statistics, see Table S7.

on lifespan is that these proteins protect worms against drug toxicity. To test this, we compared growth inhibition by metformin in wild-type and mutant *C. elegans* using a food clearance assay. *aak-2* and *skn-1* but not *daf-16* mutants showed increased sensitivity to growth inhibition by biguanides (Figures 6E, 6F, and S6D). Note that metformin-induced life extension is not *daf-16*-dependent (Onken and Driscoll, 2010). We also observed that metformin induced a similar level of *gst-4* expression in worms on *E. coli* OP50 and HT115 (+29 and +30%, respectively) even though the drug increases lifespan only with the former strain (Figure 6G). These findings further suggest that *aak-2* and *skn-1* protect worms against biguanide toxicity.

To test this further, we raised *E. coli* with or without metformin, and then transferred it to metformin-free plates with carbenicillin to prevent further growth. Carbenicillin does not affect *E. coli*-mediated effects of metformin (Figure 2D). Notably, metformin-pretreated *E. coli* caused a larger increase in mean lifespan in wild-type worms than *aak-2* worms (+48 and +29%, respectively, $p < 0.001$, Figure 6H) but not *skn-1* worms (+17 and +21%, respectively, $p < 0.0001$; Figure 6I). Moreover, extension of lifespan by blocking folate metabolism with 1 μg/ml TRI (Figure S6E) or by a folate- deficient mutant *E. coli aroD* also appeared to be partially *aak-2*-dependent (Figure S6F). These results suggest that AMPK-dependence of life extension by metformin is partly due to resistance against drug toxicity, but also partly to AMPK mediation of microbial effects on the worm. By contrast, *skn-1* activation appears to act solely by protecting against the life shortening effect of metformin.

How might SAMe levels regulate AMPK? Increased levels of SAMe can inhibit AMPK activation (Martínez-Chantar et al., 2006). To probe this we tested whether longevity induced by *sams-1* RNAi is AMPK-dependent, and this proved to be the case (Figure S6G). This suggests that metformin increases lifespan at least in part via the AMPK-activating effects of reduced SAMe levels.

Metformin Does Not Extend Lifespan on a High Glucose Diet

Metformin is a treatment for hyperglycemia caused by diabetes. We wondered whether metformin is able to provide protection against high glucose levels, which can shorten worm lifespan (Lee et al., 2009). In fact, metformin proved unable to extend the lifespan of worms supplemented with 0.25% or 1% glucose (Figures 7A and S7A; Table S7), but instead shortened lifespan. Next we tested whether high glucose affected inhibition of bacterial growth by metformin. Strikingly, glucose supplementation suppressed metformin-induced inhibition of bacterial growth (Figures S7B–S7D). This may reflect a switch from amino acid-based to glucose-based metabolism for growth, relieving the need of glucogenic amino acids (e.g., methionine) as a source of carbon. Thus, a diet high in glucose can abrogate the beneficial effects of metformin on lifespan, a finding of potential relevance to mammals.

DISCUSSION

In this study we have shown how metformin slows aging in *C. elegans* by metabolic alteration of the *E. coli* with which it is cultured. Metformin disrupts the bacterial folate cycle, leading to reduced levels of SAMe and decelerated aging in the worm.

Two Mechanisms of Action of Metformin on *C. elegans*

The effect of metformin on worm lifespan was strongly dependent upon the accompanying microbes. In the presence of some *E. coli* strains, metformin increased lifespan, whereas with other strains or in the absence of microbes it shortened lifespan. This study demonstrates that metformin has both direct

and indirect effects on *C. elegans*. Metformin (50 mM) acts directly to shorten worm lifespan, likely reflecting drug toxicity, and indirectly to increase lifespan by impairing microbial folate metabolism. The actual effect of metformin on lifespan depends on whether direct or indirect effects predominate. Given metformin-sensitive *E. coli* strains (e.g., OP50), drug treatment impairs folate metabolism and slows aging. But given metformin-resistant strains (e.g., OP50-MR), folate metabolism is less affected, the toxic effect predominates, and lifespan is shortened. It is possible that in other host organisms the capacity for metformin to slow aging is also microbiome-dependent. For example, the recent observation that metformin activates AMPK but does not increase lifespan in *Drosophila* (Slack et al., 2012) might reflect the presence of metformin-resistant microbiota.

Our findings imply that life-extending effects of metformin are not due to rescue from proliferation-mediated bacterial pathogenicity. Instead, the drug alters bacterial metabolism, leading to a state of nutritional restriction in the worm, which increases lifespan. Consistent with this, as under DR, concentrations of biguanides that increase lifespan also reduce egg laying rate (Onken and Driscoll, 2010) (Figures S1A and S1B) and reduce the rate of increase in age-specific mortality (Figures 1D and S6C) (Wu et al., 2009).

It was previously demonstrated that AMPK-dependent activation of SKN-1 is essential for metformin benefits on health span and lifespan (Onken and Driscoll, 2010). Our findings show that AMPK and SKN-1 promote resistance to biguanide toxicity, and imply it is for this reason that in their absence drug-induced life extension is not seen. However, AMPK (but not SKN-1) is also required for the full microbe-mediated life extension (Figure 6H).

Metformin Effects on Methionine Metabolism in *E. coli* and *C. elegans*

We investigated the likely bacterial target of metformin, first ruling out DHF reductase as a target (Figures S4E–S4G). Instead, metformin induction of a methyl trap, in which 5-methyl-THF accumulates, is consistent with lowered MS activity (Nijhout et al., 2004) and therefore attenuated methionine biosynthesis. Moreover, metformin also increases bacterial levels of SAMe, which is known to inhibit transcription of genes involved in methionine biosynthesis (Banerjee and Matthews, 1990). Studies in mammalian liver cells show that SAMe can act both as an allosteric activator of SAMS and a feedback inhibitor of MTHFR leading to reduced levels of methionine. In addition, increased levels of 5-methyl-THF block methyltransferases (e.g., glycine *N*-methyltransferase) (Mato et al., 2008). This provides a potential explanation for the observed rise of SAMe in addition to MS inhibition by metformin, and strongly suggest that it reduces bacterial methionine levels (Figure 7C).

Consistent with this, treating the *C. elegans*/*E. coli* system with metformin caused a 5-fold decrease in SAMe levels and a drop in the SAMe/SAH ratio in the worm. Moreover, mutation of the worm MS gene *metr-1* enhanced metformin-induced life extension, again consistent with MS inhibition in metformin-treated *E. coli*, and also with methionine restriction as a mechanism of worm life extension. The latter is further supported by the inability of metformin to extend the lifespan of *sams-1* mutant worms, which have a 65% decrease in SAMe levels (Walker et al., 2011).

Both *sams-1* RNAi and metformin increase lifespan in wild-type but not *eat-2* (DR) mutants worms, and both treatments are thought to recapitulate the effects of DR (Hansen et al., 2005; Onken and Driscoll, 2010). Indeed, metformin induces a DR-like state that, similarly to decreased levels of *sams-1* by RNAi, reduces brood size, delays reproductive timing, and increases lifespan independently of the transcription factor DAF-16/FoxO but not in *eat-2* DR mutants (Onken and Driscoll, 2010). Also, *sams-1* mRNA levels are reduced 3-fold in *eat-2* mutants (Hansen et al., 2005). Similar DR-like phenotypes, including reduced body size, were observed in our study when using phenformin (Figures S1A–S1D). Moreover, restriction of dietary methionine can extend lifespan in fruit flies and rodents (Grandison et al., 2009; Orentreich et al., 1993).

Taken with these observations, our findings suggest a potential common mechanism underlying the action of metformin, knockdown of *sams-1* and DR, which will be interesting to investigate in future studies. Potential mechanisms by which reduced SAMe might increase lifespan include reduced protein synthesis and altered fat metabolism (Ching et al., 2010; Hansen et al., 2005; Walker et al., 2011). Additionally, reduced SAMe/SAH ratio, as a measure of reduced methylation potential, could modulate lifespan via histone methylation (i.e., epigenetic effects). One possibility is that the relative abundance of metabolites such as SAMe allows the cell to assess its energy state and respond accordingly, creating a link between diet, metabolism and gene expression to modulate physiology and consequently lifespan.

Metformin and Gut Microbiota in Humans

Our findings are of potential relevance to mammalian biology and human health. Bacteria in the human gut play a central role in nutrition and host biology, and affect the risk of obesity and associated metabolic disorders such as diabetes, inflammation, and liver diseases (Cani and Delzenne, 2007). Our finding that metformin influences *C. elegans* aging by altering microbial metabolism raises the possibility that this drug might similarly influence mammalian biology by affecting microbial metabolism or composition.

Metformin is the most prescribed drug to treat T2D, with doses ranging from 500–2,500 mg/day (Scarpello and Howlett, 2008). Drug concentration in the jejunum is 30- to 300-fold higher than in the plasma in metformin recipients (Bailey et al., 2008) and concentrations above 20 mM have been detected in the intestinal lumen after administration of 850 mg metformin (Proctor et al., 2008). Interestingly, common side effects include gastrointestinal disorders (e.g., bloating and diarrhea) (Bytzer et al., 2001), reduced folate, and increased homocysteine levels (Sahin et al., 2007). Similarly, we find that metformin impairs bacterial folate metabolism and reduces host SAMe/SAH ratio.

Factors causing perturbation of the microbiome (dysbiosis), e.g., obesity, a high-fat diet, and antibiotics, often lead to metabolic dyshomeostasis in the host (Delzenne et al., 2011; Nicholson et al., 2012) e.g., due to release of proinflammatory microbial LPS into the bloodstream. Our data show that the effects of metformin are bacterial strain-dependent but independent of

LPS. One possibility is that metformin might promote a better balance of gut microbiota species. We were able to develop a metformin-resistant bacterial strain that confers benefits to the host (Figure S3F) suggesting that long-term administration of metformin could benefit the host even after treatment is ceased. Indeed, metformin administration to rats causes a change in the composition of the microbiome (Pyra et al., 2012), although it remains unclear what effect this has upon the host. Moreover, the antibiotic norfloxacin can induce alteration of mouse gut microbiome that has beneficial effects, e.g., enhanced glucose tolerance (Membrez et al., 2008).

Lowering dietary glucose can benefit humans with metabolic syndrome or T2D (Venn and Green, 2007). Diet strongly influences the metabolism of the human microbiota (Turnbaugh et al., 2009). We have found that elevated dietary glucose suppresses the effects of metformin on bacterial growth and worm lifespan. This suggests that a high-sugar diet might impair microbe-mediated benefits of metformin.

Overall, our findings point to the potential therapeutic efficacy of drugs that alter gut microbiota, particularly to prevent or treat metabolic disease (Delzenne et al., 2011). In addition, it underscores the value of *C. elegans* as a model to study host-microbe interactions.

E. coli as Food Source and Microbiome for *C. elegans*

Mammals, including humans, coexist with intestinal microbes in a relationship that includes elements of commensalism, symbiosis, and pathogenesis, and microbiota strongly influences host metabolism (Delzenne et al., 2011; Nicholson et al., 2012). Several observations suggest that in at least some respects *E. coli* could act as microbiome for *C. elegans*. Although worms can be cultured on semidefined media in the absence of *E. coli* (axenically), such media do not support normal growth and fertility. *C. elegans* seems to require live microbes for normal growth, reproduction, and aging (Lenaerts et al., 2008; Smith et al., 2008).

However, unlike microbiota and their mammalian hosts, *E. coli* is the principal food source for *C. elegans*. Studies of GFP-labeled *E. coli* imply that in late stage larvae (L4), bacterial cells are largely broken down by the pharynx prior to entering the intestine (Kurz et al., 2003), although by day 2 of adulthood intact *E. coli* are visible in the intestine (Labrousse et al., 2000). In senescent worms, *E. coli* contribute to the demise of their host, clogging the lumen of the alimentary canal and invading the intestine (Garigan et al., 2002; Labrousse et al., 2000; McGee et al., 2011). Thus, it appears that in early life *C. elegans* and *E. coli* exist in a predator-prey relationship, whereas in late life the tables are turned. But it remains possible that metabolic activity in intact or lysed *E. coli* within the worm contributes to intestinal function and host metabolism throughout life.

Presumably, *C. elegans* has evolved in the constant presence of metabolically active intestinal microbes. We postulate that, consequently, intestinal function requires their presence. Thus, it may only be possible to fully understand *C. elegans* metabolism as it operates within the *C. elegans*/*E. coli* holobiont (Zilber-Rosenberg and Rosenberg, 2008). Our account of how metformin impacts on the two organisms is consistent with this view.

EXPERIMENTAL PROCEDURES

Strains and Culture Conditions

Nematode and bacterial strains used and generated in this study are described in the Extended Experimental Procedures. Where indicated, molten NGM agar was supplemented with drugs. Axenic plates were prepared as previously described (Lenaerts et al., 2008).

Lifespan Analysis

This was performed as follows, unless otherwise indicated. Briefly, trials were initiated by transfer of L4-stage worms (day 0) on plates supplemented with 15 μM FUdR. Statistical significance of effects on lifespan was estimated using the log rank test, performed using JMP, Version 7 (SAS Institute).

GST-4::GFP Fluorescence Quantitation

Animals were raised from L1 stage on control or drug-treated plates. Quantification of GFP expression at the L4 stage was carried out using a Leica DMRXA2 epifluorescence microscope, an Orca C10600 digital camera (Hamamatsu, Hertfordshire, UK), and Volocity image analysis software (Improvision, UK). GFP intensity was measured as the pixel density in the entire cross-sectional area of each worm from which the background pixel density was subtracted (90 worms per condition).

Bacterial Growth Assay

Liquid bacterial growth was performed in microtiter plates containing the respective bacterial strain (previously grown overnight in LB and diluted 1,000-fold) and drugs in 200 μl of LB at pH 7.0. Absorbance (OD 600 nm) was measured every 5 min over an 18 hr period with shaking at 37°C using a Tecan Infinite M2000 microplate reader and Magellan V6.5 software. For colony forming unit counts, see Extended Experimental Procedures.

Bacterial Respiration

This was measured in a Clark-type oxygen electrode (Rank Brothers, Cambridge, UK) in a 1 ml stirred chamber at 37°C (Lenaerts et al., 2008).

Metabolite Analysis by LC-MS/MS

Bacterial and nematode metabolite analysis was performed as described in Extended Experimental Procedures.

Metabolomic Principal Component Analysis

Raw LC-MS/MS spectral data were uploaded into MetaboAnalyst. To avoid propensity to data overfitting, PCA analysis was used to create the 2D analysis plot.

Western Blotting

Briefly, phosphorylation of AAK-2 subunit (pAMPKα) was detected using pAMPKα (Cell Signaling) at a 1:1,000 dilution. Films were scanned and the density of each band or the entire lane was quantified by densitometry using ImageQuant TL (GE Healthcare Europe Gmb, UK).

Food Clearance Assay

The effect of biguanide compounds on *C. elegans* physiology was monitored from the rate at which 50% of the *E. coli* food suspension was consumed, as a read out for *C. elegans* growth, survival, or fecundity.

SUPPLEMENTAL INFORMATION

Supplemental Information includes Extended Experimental Procedures, seven figures, and seven tables and can be found with this article online at http://dx.doi.org/10.1016/j.cell.2013.02.035.

ACKNOWLEDGMENTS

We thank Dan Ackerman, Joy Alcedo, Nazif Alic, Caroline Araiz, Alex Benedetto, Steve Clarke, Gonçalo Correia, Monica Driscoll, Michael Murphy, Brian Onken, Matthew Piper, Bhupinder Virk, and Matthias Ziehm for useful

discussion and other help. Strains were provided by the CGC and CGSC (DBI-0742708). We acknowledge funding from the Wellcome Trust (Strategic Award), the European Union (LifeSpan) and the MRC (J003794).

Received: August 6, 2012
Revised: November 7, 2012
Accepted: February 11, 2013
Published: March 28, 2013

REFERENCES

Anisimov, V.N., Berstein, L.M., Popovich, I.G., Zabezhinski, M.A., Egormin, P.A., Piskunova, T.S., Semenchenko, A.V., Tyndyk, M.L., Yurova, M.N., Kovalenko, I.G., and Poroshina, T.E. (2011). If started early in life, metformin treatment increases lifespan and postpones tumors in female SHR mice. Aging (Albany NY) *3*, 148–157.

Bäckhed, F., Ley, R.E., Sonnenburg, J.L., Peterson, D.A., and Gordon, J.I. (2005). Host-bacterial mutualism in the human intestine. Science *307*, 1915–1920.

Bailey, C.J., Wilcock, C., and Scarpello, J.H. (2008). Metformin and the intestine. Diabetologia *51*, 1552–1553.

Banerjee, R.V., and Matthews, R.G. (1990). Cobalamin-dependent methionine synthase. FASEB J. *4*, 1450–1459.

Brenner, S. (1974). The genetics of Caenorhabditis elegans. Genetics *77*, 71–94.

Bytzer, P., Talley, N.J., Jones, M.P., and Horowitz, M. (2001). Oral hypoglycaemic drugs and gastrointestinal symptoms in diabetes mellitus. Aliment. Pharmacol. Ther. *15*, 137–142.

Cani, P.D., and Delzenne, N.M. (2007). Gut microflora as a target for energy and metabolic homeostasis. Curr. Opin. Clin. Nutr. Metab. Care *10*, 729–734.

Ching, T.T., Paal, A.B., Mehta, A., Zhong, L., and Hsu, A.L. (2010). drr-2 encodes an eIF4H that acts downstream of TOR in diet-restriction-induced longevity of C. elegans. Aging Cell *9*, 545–557.

Delzenne, N.M., and Cani, P.D. (2011). Gut microbiota and the pathogenesis of insulin resistance. Curr. Diab. Rep. *11*, 154–159.

Delzenne, N.M., Neyrinck, A.M., Backhed, F., and Cani, P.D. (2011). Targeting gut microbiota in obesity: effects of prebiotics and probiotics. Nat. Rev. Endocrinol. *7*, 639–646.

Dowling, R.J., Goodwin, P.J., and Stambolic, V. (2011). Understanding the benefit of metformin use in cancer treatment. BMC Med. *9*, 33.

Garigan, D., Hsu, A.L., Fraser, A.G., Kamath, R.S., Ahringer, J., and Kenyon, C. (2002). Genetic analysis of tissue aging in Caenorhabditis elegans: a role for heat-shock factor and bacterial proliferation. Genetics *161*, 1101–1112.

Garsin, D.A., Villanueva, J.M., Begun, J., Kim, D.H., Sifri, C.D., Calderwood, S.B., Ruvkun, G., and Ausubel, F.M. (2003). Long-lived C. elegans daf-2 mutants are resistant to bacterial pathogens. Science *300*, 1921.

Gems, D., and Riddle, D.L. (2000). Genetic, behavioral and environmental determinants of male longevity in Caenorhabditis elegans. Genetics *154*, 1597–1610.

Grandison, R.C., Piper, M.D., and Partridge, L. (2009). Amino-acid imbalance explains extension of lifespan by dietary restriction in Drosophila. Nature *462*, 1061–1064.

Hannich, J.T., Entchev, E.V., Mende, F., Boytchev, H., Martin, R., Zagoriy, V., Theumer, G., Riezman, I., Riezman, H., Knölker, H.J., and Kurzchalia, T.V. (2009). Methylation of the sterol nucleus by STRM-1 regulates Dauer larva formation in Caenorhabditis elegans. Dev. Cell *16*, 833–843.

Hansen, M., Hsu, A.L., Dillin, A., and Kenyon, C. (2005). New genes tied to endocrine, metabolic, and dietary regulation of lifespan from a Caenorhabditis elegans genomic RNAi screen. PLoS Genet. *1*, 119–128.

Hawley, S.A., Boudeau, J., Reid, J.L., Mustard, K.J., Udd, L., Mäkelä, T.P., Alessi, D.R., and Hardie, D.G. (2003). Complexes between the LKB1 tumor suppressor, STRAD alpha/beta and MO25 alpha/beta are upstream kinases in the AMP-activated protein kinase cascade. J. Biol. *2*, 28.

Ikeda, T., Yasui, C., Hoshino, K., Arikawa, K., and Nishikawa, Y. (2007). Influence of lactic acid bacteria on longevity of Caenorhabditis elegans and host defense against salmonella enterica serovar enteritidis. Appl. Environ. Microbiol. *73*, 6404–6409.

Kaeberlein, T.L., Smith, E.D., Tsuchiya, M., Welton, K.L., Thomas, J.H., Fields, S., Kennedy, B.K., and Kaeberlein, M. (2006). Lifespan extension in Caenorhabditis elegans by complete removal of food. Aging Cell *5*, 487–494.

Kau, A.L., Ahern, P.P., Griffin, N.W., Goodman, A.L., and Gordon, J.I. (2011). Human nutrition, the gut microbiome and the immune system. Nature *474*, 327–336.

Kenyon, C.J. (2010). The genetics of ageing. Nature *464*, 504–512.

Kurz, C.L., Chauvet, S., Andrès, E., Aurouze, M., Vallet, I., Michel, G.P., Uh, M., Celli, J., Filloux, A., De Bentzmann, S., et al. (2003). Virulence factors of the human opportunistic pathogen Serratia marcescens identified by in vivo screening. EMBO J. *22*, 1451–1460.

Kwon, Y.K., Lu, W., Melamud, E., Khanam, N., Bognar, A., and Rabinowitz, J.D. (2008). A domino effect in antifolate drug action in Escherichia coli. Nat. Chem. Biol. *4*, 602–608.

Labrousse, A., Chauvet, S., Couillault, C., Kurz, C.L., and Ewbank, J.J. (2000). Caenorhabditis elegans is a model host for Salmonella typhimurium. Curr. Biol. *10*, 1543–1545.

Lee, S.J., Murphy, C.T., and Kenyon, C. (2009). Glucose shortens the lifespan of C. elegans by downregulating DAF-16/FOXO activity and aquaporin gene expression. Cell Metab. *10*, 379–391.

Lenaerts, I., Walker, G.A., Van Hoorebeke, L., Gems, D., and Vanfleteren, J.R. (2008). Dietary restriction of Caenorhabditis elegans by axenic culture reflects nutritional requirement for constituents provided by metabolically active microbes. J. Gerontol. A Biol. Sci. Med. Sci. *63*, 242–252.

Maier, W., Adilov, B., Regenass, M., and Alcedo, J. (2010). A neuromedin U receptor acts with the sensory system to modulate food type-dependent effects on C. elegans lifespan. PLoS Biol. *8*, e1000376.

Mair, W., and Dillin, A. (2008). Aging and survival: the genetics of lifespan extension by dietary restriction. Annu. Rev. Biochem. *77*, 727–754.

Martínez-Chantar, M.L., Vázquez-Chantada, M., Garnacho, M., Latasa, M.U., Varela-Rey, M., Dotor, J., Santamaria, M., Martínez-Cruz, L.A., Parada, L.A., Lu, S.C., and Mato, J.M. (2006). S-adenosylmethionine regulates cytoplasmic HuR via AMP-activated kinase. Gastroenterology *131*, 223–232.

Mato, J.M., Martínez-Chantar, M.L., and Lu, S.C. (2008). Methionine metabolism and liver disease. Annu. Rev. Nutr. *28*, 273–293.

McGee, M.D., Weber, D., Day, N., Vitelli, C., Crippen, D., Herndon, L.A., Hall, D.H., and Melov, S. (2011). Loss of intestinal nuclei and intestinal integrity in aging C. elegans. Aging Cell *10*, 699–710.

Membrez, M., Blancher, F., Jaquet, M., Bibiloni, R., Cani, P.D., Burcelin, R.G., Corthesy, I., Macé, K., and Chou, C.J. (2008). Gut microbiota modulation with norfloxacin and ampicillin enhances glucose tolerance in mice. FASEB J. *22*, 2416–2426.

Nicholson, J.K., Holmes, E., Kinross, J., Burcelin, R., Gibson, G., Jia, W., and Pettersson, S. (2012). Host-gut microbiota metabolic interactions. Science *336*, 1262–1267.

Nijhout, H.F., Reed, M.C., Budu, P., and Ulrich, C.M. (2004). A mathematical model of the folate cycle: new insights into folate homeostasis. J. Biol. Chem. *279*, 55008–55016.

Onken, B., and Driscoll, M. (2010). Metformin induces a dietary restriction-like state and the oxidative stress response to extend C. elegans Healthspan via AMPK, LKB1, and SKN-1. PLoS ONE *5*, e8758.

Orentreich, N., Matias, J.R., DeFelice, A., and Zimmerman, J.A. (1993). Low methionine ingestion by rats extends lifespan. J. Nutr. *123*, 269–274.

Ottaviani, E., Ventura, N., Mandrioli, M., Candela, M., Franchini, A., and Franceschi, C. (2011). Gut microbiota as a candidate for lifespan extension: an ecological/evolutionary perspective targeted on living organisms as metaorganisms. Biogerontology *12*, 599–609.

Pierotti, M.A., Berrino, F., Gariboldi, M., Melani, C., Mogavero, A., Negri, T., Pasanisi, P., and Pilotti, S. (2012). Targeting metabolism for cancer treatment and prevention: metformin, an old drug with multi-faceted effects. Oncogene. http://dx.doi.org/10.1038/onc.2012.181.

Proctor, W.R., Bourdet, D.L., and Thakker, D.R. (2008). Mechanisms underlying saturable intestinal absorption of metformin. Drug Metab. Dispos. *36*, 1650–1658.

Pyra, K.A., Saha, D.C., and Reimer, R.A. (2012). Prebiotic fiber increases hepatic acetyl CoA carboxylase phosphorylation and suppresses glucose-dependent insulinotropic polypeptide secretion more effectively when used with metformin in obese rats. J. Nutr. *142*, 213–220.

Sahin, M., Tutuncu, N.B., Ertugrul, D., Tanaci, N., and Guvener, N.D. (2007). Effects of metformin or rosiglitazone on serum concentrations of homocysteine, folate, and vitamin B12 in patients with type 2 diabetes mellitus. J. Diabetes Complications *21*, 118–123.

Saiki, R., Lunceford, A.L., Bixler, T., Dang, P., Lee, W., Furukawa, S., Larsen, P.L., and Clarke, C.F. (2008). Altered bacterial metabolism, not coenzyme Q content, is responsible for the lifespan extension in Caenorhabditis elegans fed an Escherichia coli diet lacking coenzyme Q. Aging Cell *7*, 291–304.

Scarpello, J.H., and Howlett, H.C. (2008). Metformin therapy and clinical uses. Diab. Vasc. Dis. Res. *5*, 157–167.

Slack, C., Foley, A., and Partridge, L. (2012). Activation of AMPK by the putative dietary restriction mimetic metformin is insufficient to extend lifespan in Drosophila. PLoS ONE *7*, e47699.

Smith, E.D., Kaeberlein, T.L., Lydum, B.T., Sager, J., Welton, K.L., Kennedy, B.K., and Kaeberlein, M. (2008). Age- and calorie-independent lifespan extension from dietary restriction by bacterial deprivation in Caenorhabditis elegans. BMC Dev. Biol. *8*, 49.

Storelli, G., Defaye, A., Erkosar, B., Hols, P., Royet, J., and Leulier, F. (2011). Lactobacillus plantarum promotes Drosophila systemic growth by modulating hormonal signals through TOR-dependent nutrient sensing. Cell Metab. *14*, 403–414.

Turnbaugh, P.J., Ridaura, V.K., Faith, J.J., Rey, F.E., Knight, R., and Gordon, J.I. (2009). The effect of diet on the human gut microbiome: a metagenomic analysis in humanized gnotobiotic mice. Sci. Transl. Med. *1*, 6ra14.

Venn, B.J., and Green, T.J. (2007). Glycemic index and glycemic load: measurement issues and their effect on diet-disease relationships. Eur. J. Clin. Nutr. *61*(*Suppl 1*), S122–S131.

Virk, B., Correia, G., Dixon, D.P., Feyst, I., Jia, J., Oberleitner, N., Briggs, Z., Hodge, E., Edwards, R., Ward, J., et al. (2012). Excessive folate synthesis limits lifespan in the C. elegans: E. coli aging model. BMC Biol. *10*, 67.

Walker, A.K., Jacobs, R.L., Watts, J.L., Rottiers, V., Jiang, K., Finnegan, D.M., Shioda, T., Hansen, M., Yang, F., Niebergall, L.J., et al. (2011). A conserved SREBP-1/phosphatidylcholine feedback circuit regulates lipogenesis in metazoans. Cell *147*, 840–852.

Wu, D., Rea, S.L., Cypser, J.R., and Johnson, T.E. (2009). Mortality shifts in Caenorhabditis elegans: remembrance of conditions past. Aging Cell *8*, 666–675.

Zilber-Rosenberg, I., and Rosenberg, E. (2008). Role of microorganisms in the evolution of animals and plants: the hologenome theory of evolution. FEMS Microbiol. Rev. *32*, 723–735.

EXTENDED EXPERIMENTAL PROCEDURES

Nematode and Bacterial Strains

Nematode strains used include: wild-type (N2), CL2166 *dvIs19 [pAF15 (gst-4::GFP::NLS)]*, EU31 *skn-1(zu135)/nT1 [unc-?(n754) let-?]*, GA1001 *aak-2(ok524)*, GA1112 *aak-2(ok524); dvIs19[pAF15 (gst-4::GFP::NLS)]*, GR1307 *daf-16(mgDf50)*, RB755 *metr-1(ok521)* and RB2240 *sams-1(ok3033)*.

E. coli strains used include: BL21-Gold(DE3) (Studier et al., 1990), CS180 [rfa+](Pradel et al., 1992), CS2429 [rfaC$^-$ of CS180] (Zhang et al., 2006) kindly provided by Joy Alcedo, GD1 *ubiG*, GD1::pAHG (*ubiG*$^+$) (Larsen and Clarke, 2002) kindly provided by Catherine Clarke, HB101 (Boyer and Roulland-Dussoix, 1969), HT115 [rnc14::ΔTn10 λ(DE3) of W3110], HT115(DE3) *aroD717::*IS*1* (Virk et al., 2012), MG1655 (Jensen, 1993), OP50 (Brenner, 1974), OP50-MR (metformin-resistant, made in this study), OP50-R26, containing the R26 P-group plasmid which confers resistance to multiple antibiotics (Stanisich and Ortiz, 1976) (made by mating with C600 R26) (Villarroel et al., 1983; Virk et al., 2012) and OP50-tmpR (made in this study). *Bacillus subtilis* PY79 (Garsin et al., 2003) was kindly provided by Danielle Garsin.

Nematode Culture Conditions

Nematode growth media was prepared as described (Brenner, 1974). Where indicated molten agar was supplemented with phenformin (1.5, 3, 4.5 mM), metformin (25, 50, 100 mM), carbenicillin (120 μM), trimethoprim (TMP 0.1, 0.2, 0.5, 1 μg/ml), D-glucose (0.25, 1%). For the carbenicillin treatment of bacteria, 80 μl of 500 mM carbenicillin stock solution was added to the plates after 48 hr of bacterial growth as described (Garigan et al., 2002). For the UV treatment of bacteria, 80 μl of *E. coli* OP50 suspension was seeded onto an NGM plate and left to grow at 20°C overnight. Plates were then irradiated for 30 min on a UV Stratalinker 2400 (Stratagene) containing bulbs irradiating at 254 nm. Plates were further irradiated for 5 min on the day of transfer. Axenic plates were prepared as previously described (Lenaerts et al., 2008). Briefly, axenic solid media is composed of 3% peptone, 3% yeast extract and 0.05% hemoglobin (diluted in 0.1 M KOH). Hemoglobin is added to the autoclaved molten agar with constant stirring. Plates are kept at 4°C and in the dark. Bacterial deprivation is performed with NGM plates in the absence of *E. coli*, as previously described (Kaeberlein et al., 2006). Plates at pH = 6.0-6.5 were used and the pH was confirmed using pH indicator strips (note that metformin effects on *E. coli* are pH-sensitive; see Figures S2G and S2H). All chemicals were purchased from Sigma-Aldrich.

Lifespan and Mortality Analysis

Lifespan measurements were performed as follows, unless indicated otherwise in Supplementary Tables. Axenic worm eggs were obtained using alkaline hypochlorite treatment of gravid adult hermaphrodites. These were then placed onto plates containing the test bacterial strain and maintained at 20°C for 2 generations. Lifespan measurements were initiated by transfer of L4-stage worms (day 0) to plates containing bacteria grown in the presence or absence of treatment for 96 hr. Worms were transferred to fresh plates every 4 days until day 12, to plates containing bacteria grown for 96 hr at 20°C. To prevent progeny development plates were supplemented with 5-fluoro-2′-deoxyuridine (FUdR, 15 μM) on the day prior to use, unless indicated otherwise.

For the survival assays involving metformin-pretreatment of *E. coli*, 4-day old *E. coli* grown on control or metformin-treated plates were transferred using a 24 mm cell culture scraper (TPP®, Switzerland) to NGM plates supplemented with carbenicillin and FUdR. Worms growing on *E. coli* OP50 for 2 generations were transferred at the L4 stage to start the lifespan. Worms were transferred every 2 days to fresh plates containing metformin-pretreated or control *E. coli*. For axenic, bacterial deprivation and UV-treated bacteria lifespans, bleached eggs were placed onto UV-irradiated bacterial plates. This allows worm development and prevents bacterial contamination. At the L4 stage, worms were transferred to each of the treatment/condition plates containing FUdR (15 μM) and transferred every 2 days to new fresh plates. For RNAi experiments, eggs were added to plates seeded with the bacteria expressing double-stranded RNA to *sams-1*. Lifespans were initiated by the transfer of L4 stage worms to new fresh plates containing FUdR (10 μM). Worms were transferred to new fresh plates every 2 days until days 12 and then every 5 days. Worms that showed severe vulva protrusion or bagging were censored. Survival was monitored at regular time points and worms scored as dead if they did not show any movement when prodded with a platinum wire. Statistical significance of effects on lifespan was estimated using the log rank test, performed using JMP, Version 7 (SAS Institute). The mortality plots were defined as log(−log(1−n/event(x)/n.risk(x)/time(x)−time(x-1))) over time (x). Smoothing was applied using a sliding window approach.

RNA-Mediated Interference Clones

RNAi by feeding was performed as previously described (Kamath et al., 2001). RNAi *E. coli* feeding clones were derived from the Ahringer RNAi Library, kindly provided by Steven Nurrish.

Generation of Bacterial Strains OP50R and OP50-tmpR

E. coli OP50 was grown on NGM plates containing an initial metformin concentration of 50 mM. Positive clones were transferred to fresh plates containing increasing concentrations of metformin up to 300 mM. To favor selection of metformin-resistant bacteria, the plates were incubated at 25°C. When it was ensured that bacterial growth was not compromised, the growth temperature was shifted to 37°C. For the generation of *E. coli* OP50 TMP-resistant strain, a fragment of 349 bp comprising the trimethoprim (TMP) resistant cassette was PCR-amplified from plasmid pEAK16_GFP (kindly provided by Brian Seed) using

primers 5′-CCAGCAACGCAAGCTAGAGTT and 5′-GTCCTCCTTACCAGAAATTTATCC and cloned into pGEM-T Easy Vector System (Promega). Resulting plasmid (pNV6) was transformed into *E. coli* OP50 competent cells by standard procedure. Transformants were plated in LB (Luria-Bertani) agar containing ampicillin (100 mg/ml) and trimethoprim (50 μg/ml) and incubated at 37°C for 12–14 hr. Positive clones were selected and the presence of plasmid pNV6 was verified by plasmid extraction and sequencing.

Genome Sequencing of Bacterial Strains OP50 and OP50R

An Illumina MiSeq machine was used to generate 150bp paired-end reads from the genomes of the OP50 parental (5.86 million reads) and the resistant (4.06 million reads) strains. Fastq files were aligned to the *Escherichia coli* B strain REL606 genome using the Burrows-Wheeler Aligner (BWA) (Li and Durbin, 2009) and the median coverage for the parental strain was 325x and 189x for the resistant strain. VarScan (Koboldt et al., 2012) was used to identify SNP's and indels. There were eight SNP's with variant frequency above 90% in the resistant strain but not present (i.e., variant frequency less than 1%) in the parental or reference genome. Two of these SNP's result in single amino acid changes in the genes argG and glyA (which is a serine hydroxymethyltransferase involved in folate-mediated one-carbon metabolism).

Fecundity, Size, and Development Time Measurements

Synchronized L1 larvae were obtained by bleaching of gravid adults and left overnight in M9 solution. Larvae were then raised on control and respective drug treatments until reaching the L4 stage. For developmental assays the proportion of worms that had reached the L4 stage was determined at 12 hr intervals. Worm length was measured at the L4 stage using a Leica RXA2 compound microscope and the image analysis application Volocity (Improvision, UK). For brood size and fecundity timing assays, starting with L4 worms, animals were transferred daily to fresh new plates during the reproductive period and progeny numbers counted. All data present the average of at least 3 independent trials.

GST-4::GFP Fluorescence Quantitation

CL2166 *dvIs19 [pAF15(gst-4::GFP::NLS)]* and GA1112 *aak-2(ok524); dvIs19 [pAF15 (gst-4::GFP::NLS)]* animals were raised from synchronized L1 larvae in control, 50 mM metformin and 4.5 mM phenformin plates. L4 worms were picked and placed in a drop of 0.06% levamisole and observed under a 10x objective. Quantification of GFP expression from the transgene was carried out using a Leica DMRXA2 microscope using a GFP filter cube (excitation: 470/40 nm; emission: 525/50 nm), an Orca C10600 digital camera (Hamamatsu) and Volocity image analysis software (Improvision, UK). GFP intensity was measured as the pixel density in the entire cross sectional area of each worm from which the background pixel density was subtracted (90 worms per condition).

Bacterial Growth Measurements

Liquid bacterial growth was performed in microtiter plates containing the respective bacterial strain (previously grown overnight in LB and diluted 1,000-fold) and drugs in 200 μl of LB at pH = 7.0. The absorbance (OD 600 nm) was measured every 5 min for an 18 hr incubation period with regular shaking at 37°C using a Tecan Infinite M2000 microplate reader and Magellan V6.5 software. Data analysis was performed on 3 or more replicate trials for each condition. Values for bar graphs are taken from OD_{600} values at 18 hr of growth. Each bacterial strain was previously grown overnight in LB at 37°C (200 rpm).

For colony forming unit counts, bacteria growing in control or treatments plates was scraped from the plate, resuspended in M9 and normalized to an $OD_{600} = 0.1$. Each sample was diluted to $1x10^{-5}$ and 5 μl streaked on an LB agar plate followed by incubation at 37°C for 16 hr. The number of colonies formed was scored for each condition.

Bacterial Respiration

Bacterial respiration was measured in a Clark-type oxygen electrode (Rank Brothers, Cambridge, UK) in a 1 ml stirred chamber set at 37°C (Lenaerts et al., 2008). The electrode was calibrated using air-saturated H_2O assuming 406 nmol O_2/ml at 37°C. Bacteria grown on plates ±50 mM metformin were resuspended in LB medium (OD_{600} ~0.25–0.35), and oxygen consumption was measured in the presence of exogenously added 50 mM metformin or H_2O carrier. Oxygen concentration was plotted as a function of time, and the slope of the linear part of the graph was used to estimate the oxygen consumption rate.

Sample Preparation for Metabolite Analysis

Bacteria: 4-day old bacterial lawns growing on control and metformin plates were washed from the plates using M9. The bacteria was then centrifuged at 4°C, 4,000 rpm for 20 min. The supernatant was discarded and the bacterial pellet kept at −80°C until further analysis. Worms: Approximately 600 synchronized 4-day adult worms per biological replicate from both control and metformin plates were collected. Worms were washed three times using M9 to remove bacteria and frozen at −70°C until further analysis. Test conditions for metabolite analysis were similar to those for lifespan assays at day 4. At least 3 biological replicates from each type of bacteria/worm strain and drug treatment were collected for each measurement. Buffer containing 20 mM ammonium acetate, 0.1% ascorbic acid, 0.1% citric acid and 100 mM DTT at pH 7.0 was added to cells. Suspensions were sonicated for 10 s using a hand-held sonicator at 40% amplitude for bacteria and 60% for *C. elegans*. Protein was removed by precipitation by addition of

2 sample volume of acetonitrile, mixing for two minutes and centrifugation for 15 min at 12,000 g and 4°C. Supernatants were transferred to fresh tubes, lyophilized and stored at −80°C prior to analysis.

Folate Analysis in Bacteria and *C. elegans* by LC-MS/MS

Lyophilized samples were resuspended in 50 μl water (milli-Q) and centrifuged for 5 min at 12,000 g at 4°C. Supernatants were transferred to glass sample vials for LC-MS/MS analysis. Metabolites were resolved by reversed-phase chromatography (Luna C18 column; 150 mm x 2.0 mm; 5 μm bead size; Phenomenex, UK) using a 2795XE high performance liquid chromatography unit with solvent divert valve (Waters Corporation, UK). The HPLC was coupled to a MicroMass Quattro triple quadrupole tandem mass spectrometer (Waters Corporation, UK) operating in negative-ion mode using the following settings: capillary 3.54 kV, source temperature 150°C, desolvation temperature 350°C, cone gas flow rate 25 L/h and desolvation gas flow rate 950 L/h. Folates were measured by multiple reaction monitoring (MRM) with optimized cone voltage and collision energy for precursor and product ions as previously described (Garratt et al., 2005).

Analysis of S-Adenosylmethionine and S-Adenosylhomocysteine

Quantification of SAMe and SAH was performed as described previously (Burren et al., 2006).

Metabolomic Principal Component Analysis

Raw LC-MS/MS spectral data from 3 biological replicates of each condition was uploaded into MetaboAnalyst (Xia and Wishart, 2011). Missing values were replaced by default by low signal intensity values (below detection limit). Row-wise normalization was achieved by sample normalization prior to analysis. Column-wise normalization was achieved using Pareto scaling in order to produce a typical ‘‘bell curve’’ shape of transformed data. To avoid propensity to data overfitting, PCA analysis was used to create the 2D analysis plot. The dendrogram plot was obtained through clustering of the data using the Ward method.

Western Blotting

Eggs were placed in 0, 25, 50 and 100 mM metformin plates and allowed to develop in the presence of drug until reaching 1-day adults. Additionally, L4 wild-type were placed on 0, 1.5, 3 and 4.5 mM phenformin plates for 2 days or in 50 mM metformin for 8 and 16 days. The worms were then washed 3 times using M9 to remove the bacteria and frozen at −70°C. Phospho-safe lysis buffer (Pierce) was supplemented with protease inhibitors (Roche). The lysates were homogenized using a Bioruptor (Cosmo Bio Co., Ltd., Tokyo, Japan) in 2 ml microcentrifuge tubes and then centrifuged at 16,000 g for 30 min at 4°C. The supernatants were then assayed for protein concentration using the Bradford assay. 30 mg of protein extracts were run on SDS-PAGE gel and transferred onto nitrocellulose membrane. Phosphorylation of AAK-2 subunit (pAMPKα) was detected using a rabbit antibody specific to pAMPKα (cell signaling) at a 1:1,000 dilution. As a loading control, β-actin protein was used with mouse anti-actin at a 1:5,000 dilution (Santa-Cruz Biotechnology). Blots were developed using the SuperSignal West Pico chemiluminescent substrate (Perbio Sciences). Films were scanned and the density of each band or the entire lane was quantified by densitometry using ImageQuant TL (GE Healthcare Europe GmbH).

Food Clearance Assay

The effect of biguanide compounds on *C. elegans* physiology was monitored by the rate at which the 50% of the *E. coli* food suspension was consumed, as a read out for *C. elegans* growth, survival or fecundity (Voisine et al., 2007). Approximately 20–30 L1 age-synchronized animals per 10 μl of S-media were added to an *E. coli* suspension at a final OD_{600} of 0.8. Microtiter plates containing animals and drugs were incubated at 25°C (200 rpm). OD_{600} was measured daily using a Tecan Infinite M2000 microplate reader and Magellan V6.5 software. Data analysis was performed on 3 or more biological replicates for each condition.

SUPPLEMENTAL REFERENCES

Boyer, H.W., and Roulland-Dussoix, D. (1969). A complementation analysis of the restriction and modification of DNA in *Escherichia coli*. J. Mol. Biol. *41*, 459–472.

Burren, K.A., Mills, K., Copp, A.J., and Greene, N.D. (2006). Quantitative analysis of s-adenosylmethionine and s-adenosylhomocysteine in neurulation-stage mouse embryos by liquid chromatography tandem mass spectrometry. J. Chromatogr. B Analyt. Technol. Biomed. Life Sci. *844*, 112–118.

Garratt, L.C., Ortori, C.A., Tucker, G.A., Sablitzky, F., Bennett, M.J., and Barrett, D.A. (2005). Comprehensive metabolic profiling of mono- and polyglutamated folates and their precursors in plant and animal tissue using liquid chromatography/negative ion electrospray ionisation tandem mass spectrometry. Rapid Commun. Mass Spectrom. *19*, 2390–2398.

Jensen, K.F. (1993). The *Escherichia coli* K-12 ‘‘wild types’’ W3110 and MG1655 have an rph frameshift mutation that leads to pyrimidine starvation due to low pyrE expression levels. J. Bacteriol. *175*, 3401–3407.

Kamath, R.S., Martinez-Campos, M., Zipperlen, P., Fraser, A.G., and Ahringer, J. (2001). Effectiveness of specific RNA-mediated interference through ingested double-stranded RNA in Caenorhabditis elegans. Genome Biol. *2*, RESEARCH0002.

Kim, Y.B. (2009). Improved trimethoprim-resistance cassette for prokaryotic selections. J. Biosci. Bioeng. *108*, 441–445.

Koboldt, D.C., Zhang, Q., Larson, D.E., Shen, D., McLellan, M.D., Lin, L., Miller, C.A., Mardis, E.R., Ding, L., and Wilson, R.K. (2012). VarScan 2: somatic mutation and copy number alteration discovery in cancer by exome sequencing. Genome Res. *22*, 568–576.

Larsen, P.L., and Clarke, C.F. (2002). Extension of life-span in *Caenorhabditis elegans* by a diet lacking coenzyme Q. Science *295*, 120–123.

Lee, G.D., Wilson, M.A., Zhu, M., Wolkow, C.A., de Cabo, R., Ingram, D.K., and Zou, S. (2006). Dietary deprivation extends lifespan in *Caenorhabditis elegans*. Aging Cell *5*, 515–524.

Li, H., and Durbin, R. (2009). Fast and accurate short read alignment with Burrows-Wheeler transform. Bioinformatics *25*, 1754–1760.

Pradel, E., Parker, C.T., and Schnaitman, C.A. (1992). Structures of the rfaB, rfaI, rfaJ, and rfaS genes of *Escherichia coli* K-12 and their roles in assembly of the lipopolysaccharide core. J. Bacteriol. *174*, 4736–4745.

Stanisich, V.A., and Ortiz, J.M. (1976). Similarities between plasmids of the P-incompatibility group derived from different bacterial genera. J. Gen. Microbiol. *94*, 281–289.

Studier, F.W., Rosenberg, A.H., Dunn, J.J., and Dubendorff, J.W. (1990). Use of T7 RNA polymerase to direct expression of cloned genes. Methods Enzymol. *185*, 60–89.

Villarroel, R., Hedges, R.W., Maenhaut, R., Leemans, J., Engler, G., Van Montagu, M., and Schell, J. (1983). Heteroduplex analysis of P-plasmid evolution: the role of insertion and deletion of transposable elements. Mol. Gen. Genet. *189*, 390–399.

Voisine, C., Varma, H., Walker, N., Bates, E.A., Stockwell, B.R., and Hart, A.C. (2007). Identification of potential therapeutic drugs for Huntington's disease using *Caenorhabditis elegans*. PLoS ONE *2*, e504.

Xia, J., and Wishart, D.S. (2011). Web-based inference of biological patterns, functions and pathways from metabolomic data using MetaboAnalyst. Nat. Protoc. *6*, 743–760.

Zhang, P., Snyder, S., Feng, P., Azadi, P., Zhang, S., Bulgheresi, S., Sanderson, K.E., He, J., Klena, J., and Chen, T. (2006). Role of N-acetylglucosamine within core lipopolysaccharide of several species of gram-negative bacteria in targeting the DC-SIGN (CD209). J. Immunol. *177*, 4002–4011.

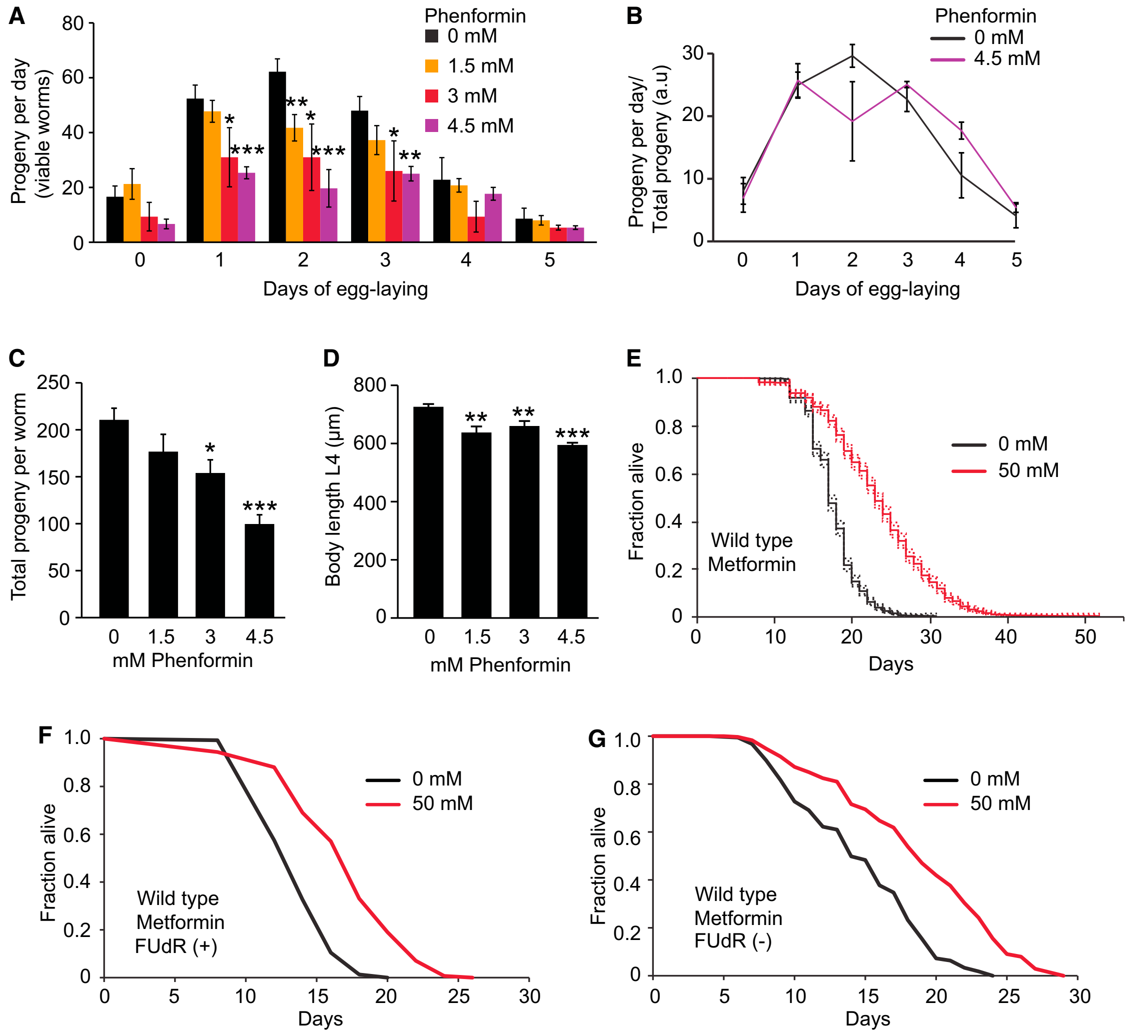

Figure S1. Effects of Biguanides on Fecundity, Growth, and Lifespan, Related to Figure 1

(A) Dose-dependent reduction in daily fecundity by phenformin.

(B) Effect of phenformin on daily fecundity as a proportion of total fecundity. Note the apparent, slight reproductive delay.

(C) Dose-dependent reduction in brood size by phenformin.

(D) Phenformin causes a reduction in body length (L4 stage).

(E) Metformin robustly increases lifespan when administered from early adulthood onward. Combined data for all survival assays performed, and corresponding to mortality data in Figure 1D (dots represent confidence intervals). We also compared effects of metformin administered for 2 generations prior to the start of the survival assay with administration from the L4 stage onward, and detected no difference (Table S1).

(F and G) Effects of metformin on lifespan are not affected by FUdR.

For statistics see Table S1. Error bars, SEM. *$p < 0.05$; **$p < 0.01$; ***$p < 0.001$.

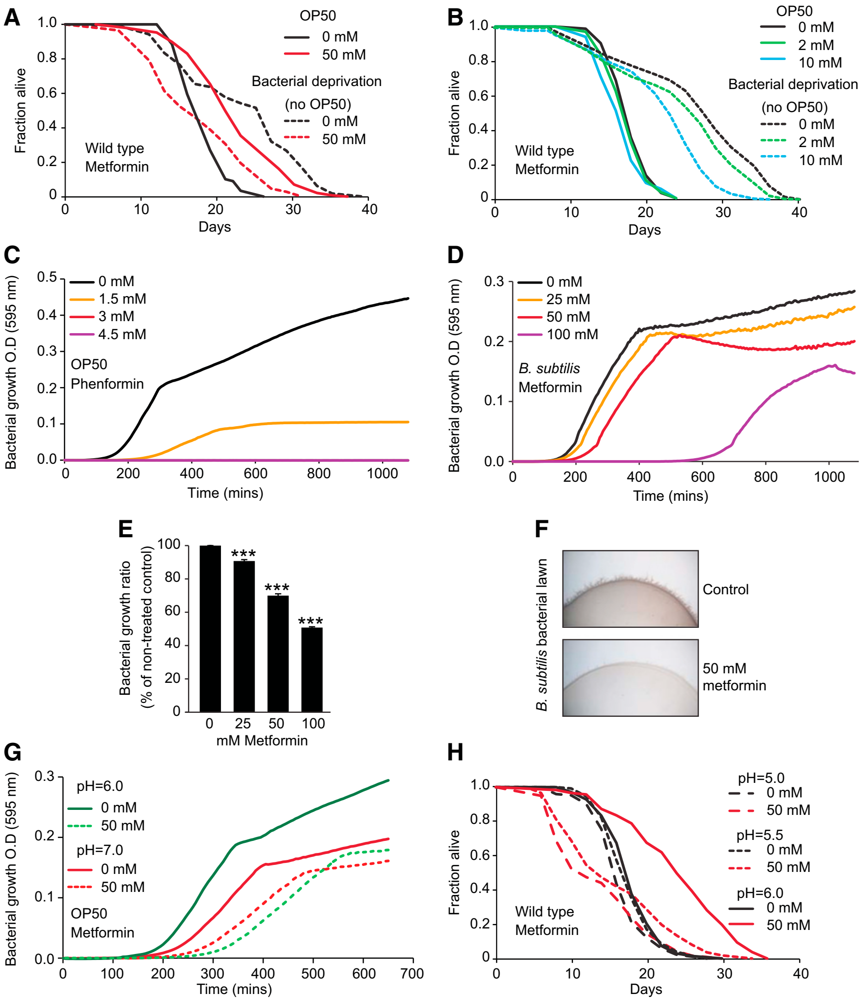

Figure S2. Biguanides Inhibit Bacterial Growth, Related to Figure 2

(A and B) Metformin shortens lifespan in the absence of bacteria (bacterial deprivation on NGM plates) (Kaeberlein et al., 2006; Lee et al., 2006).

(C) Dose-dependent inhibition of *E. coli* OP50 growth by phenformin (LB liquid media, 18 hr period).

(D–E) Dose dependent inhibition of growth of *Bacillus subtilis* by metformin (LB liquid media, 18 hr period).

(F) Metformin causes altered bacterial lawn morphology in *Bacillus subtilis*.

(G) Inhibition of bacterial growth by metformin is more marked at pH = 6.0 than at pH = 7.0.

(H) Effects of metformin on *C. elegans* lifespan are pH-sensitive.

See Table S2 for statistics. Error bars, SEM. *p < 0.05; **p < 0.01; ***p < 0.001.

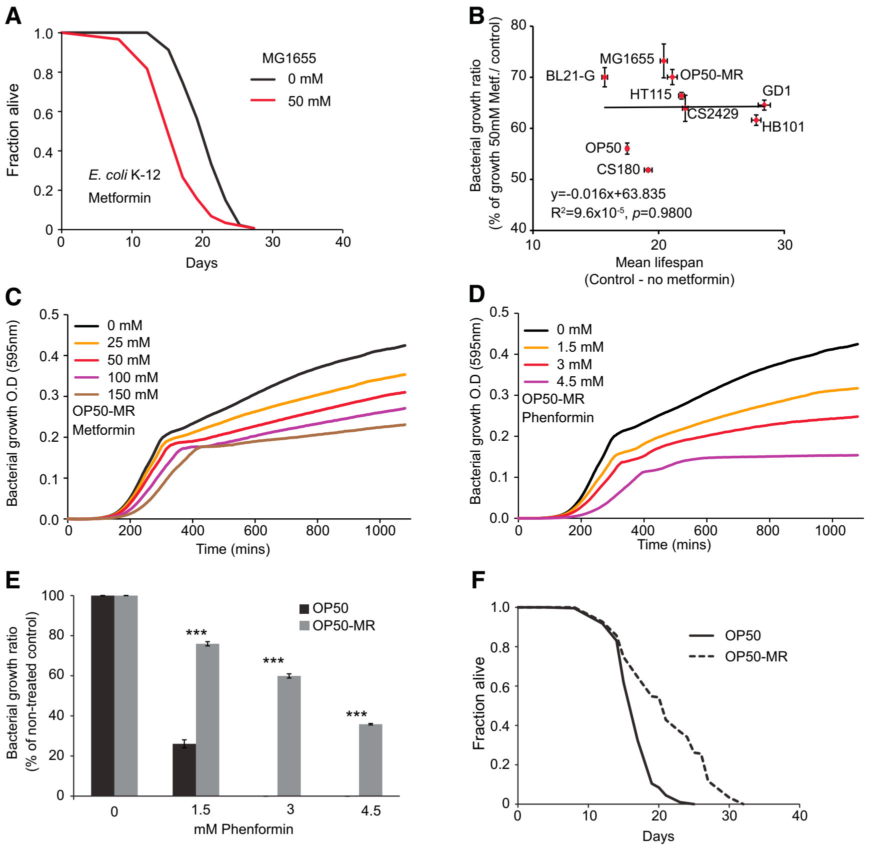

Figure S3. Effects of Metformin and Phenformin on Bacterial Growth and Lifespan of Various Bacterial Strains, Related to Figure 3

(A) Metformin shortens worm lifespan in the presence of the wild-type *E. coli* K-12 strain MG1655.

(B) *E. coli* sensitivity to growth inhibition by metformin shows no correlation with *E. coli* effects on wild-type worm lifespan.

(C) *E. coli* OP50-MR is resistant to growth inhibition by metformin compared to OP50 parent strain (c.f. Figure 2F).

(D) *E. coli* OP50-MR is resistant to growth inhibition by phenformin compared to OP50 parent strain.

(E) Relative resistance to growth inhibition by phenformin of *E. coli* OP50 (black) and OP50-MR (gray).

(F) OP50-MR increases lifespan in the absence of metformin.

For statistics see Table S3. Error bars, SEM. *p < 0.05; **p < 0.01; ***p < 0.001.

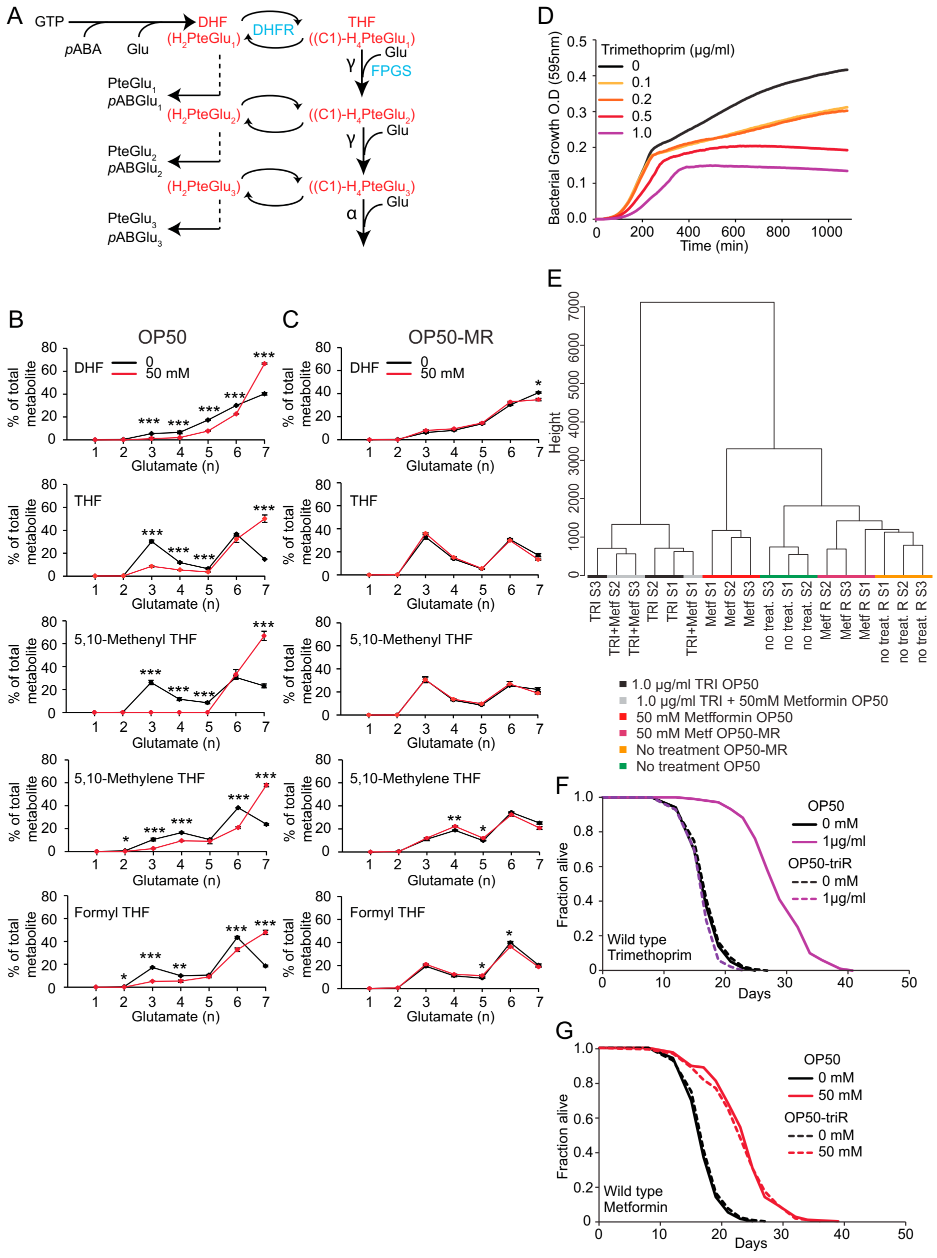

Figure S4. Metformin and Trimethoprim Alter Bacterial Metabolism and Extend *C. elegans* Lifespan by Common Mechanisms, Related to Figure 4

(A) Diagram showing folate synthesis, one-carbon substitution, polyglutamylation and catabolism in *E. coli* (adapted from Kwon et al., 2008). Blue, enzymes involved in these reactions: FPGS, folylpolyglutamate synthetase; DHFR, dihydrofolate reductase. Red: DHF, dihydrofolate; THF, tetrahydrofolate. Black: *p*ABA, *p*-aminobenzoic acid; Glu, glutamate; GTP, guanosine triphosphate; PteGlu, pteroylmonoglutamic acid; *p*ABGlu, *p*-aminobenzoyl-L-glutamate.

(B) Folate polyglutamylation profiles of detectable folate metabolites of *E. coli* OP50 grown in the presence or absence of metformin. Metformin strongly affects polyglutamylation levels of all folates detected.

(C) Folate polyglutamylation profiles of detectable folate metabolites of *E. coli* OP50-MR bacteria grown in the presence or absence of metformin (note the absence of effects in most cases).

(D) Trimethoprim (TRI) acts as a bacteriostatic antibiotic to delay bacterial growth in a dose-dependent manner.

(E) Hierarchical cluster analysis of metabolites of TRI and metformin-treated OP50 and OP50-MR using the Ward method. Metabolite clusters from the TRI+metformin condition or TRI alone are indistinguishable from each other. Metformin treatment of OP50-MR creates a cluster of metabolites similar to OP50-MR and OP50 in the absence of treatment and distinct from OP50 treated with metformin.

(F) TRI does not extend worm lifespan in the presence of a TRI-resistant strain of *E. coli* OP50 (OP50-triR) expressing the type IIa DHF reductase (Kim, 2009).

(G) TRI-resistance in *E. coli* has no effect on induction of life extension by metformin.

For statistics see Table S4. Error bars, SEM. $^{*}p < 0.05$; $^{**}p < 0.01$; $^{***}p < 0.001$.

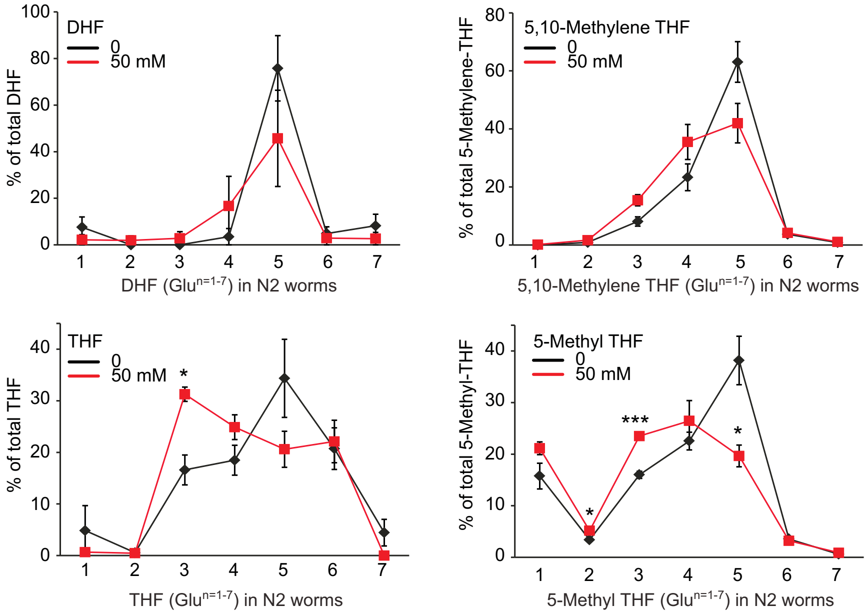

Figure S5. Folate Polyglutamylation Profiles of Detectable Folate Metabolites of Wild-Type Worms Grown in the Presence or Absence of Metformin, Related to Figure 5

Error bars, SEM of at least 3 independent biological replicates. *$p < 0.05$; **$p < 0.01$; ***$p < 0.001$.

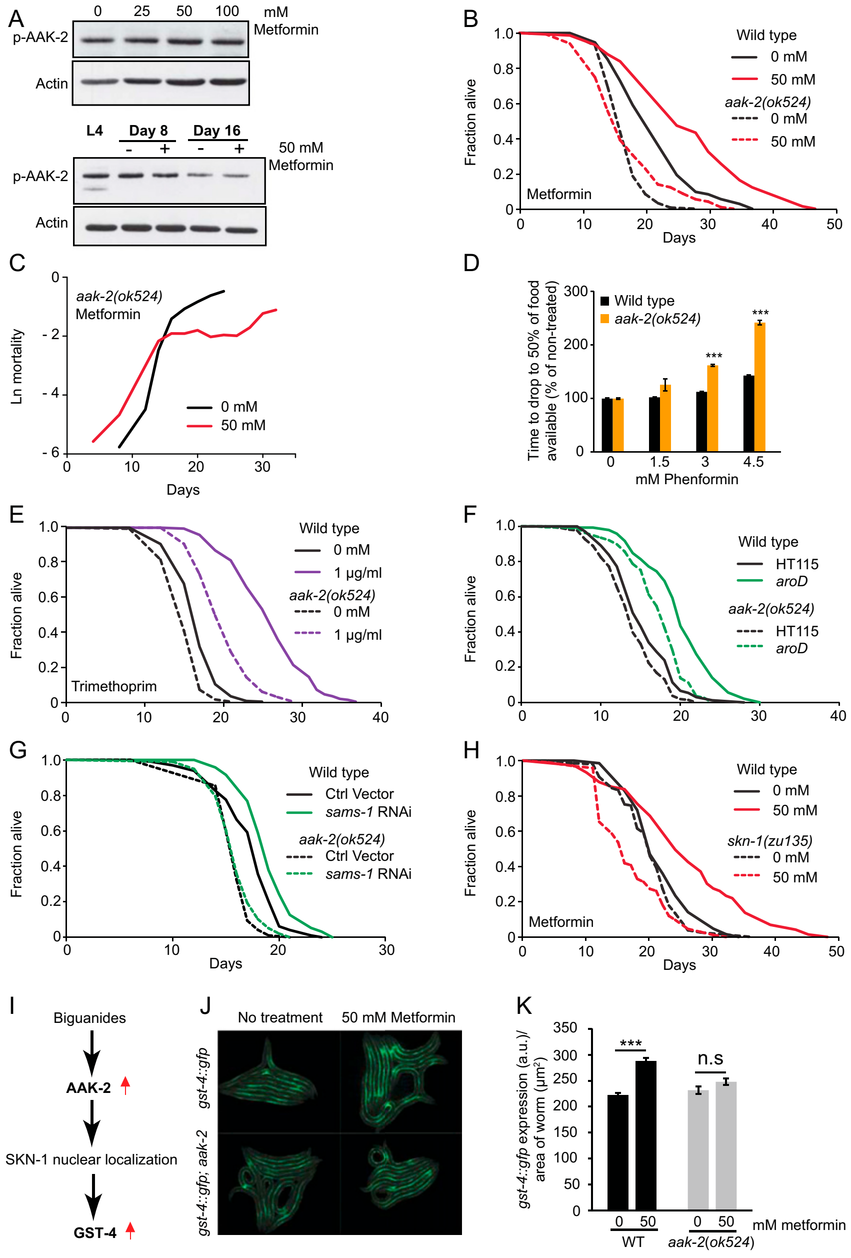

Figure S6. Role of AMP Kinase and SKN-1 in Metformin-Induced Longevity, Related to Figure 6

(A) Metformin does not detectably alter pAAK-2 levels in 1-day adults when developing in the presence of different drug concentrations or in day-8 and day-16 adults.

(B) Metformin does not significantly increase lifespan in AMPK-deficient *aak-2(ok524)* mutant worms.

(C) Metformin decreases the exponential increase in age-related mortality in AMPK-deficient *aak-2(ok524)* mutant worms, but increases initial mortality.

(D) AMPK-deficient *aak-2(ok524)* mutant worms are hypersensitive to growth inhibition by phenformin as measured using the food clearance assay (Voisine et al., 2007).

(E) TMP does extend lifespan in *aak-2(ok524)* mutant worms, though the magnitude of the extension is slightly reduced.

(F) The folate-deficient (*aroD*) mutant *E. coli* (Virk et al., 2012) does extend lifespan in *aak-2(ok524)* mutants, though the magnitude of the extension is slightly reduced.

(G) *sams-1* RNAi does not extend lifespan in *aak-2(ok524)* (AMPK-deficient) mutant worms.

(H) Metformin shortens lifespan in *skn-1(zu135)* mutant worms.

(I) Diagram showing AMPK-dependent expression of SKN-1 gene targets such as *gst-4* by biguanides; after Onken and Driscoll (2010).

(J and K) Metformin induces *gst-4::gfp* expression in an AMPK dependent fashion, consistent with a previous report (Onken and Driscoll, 2010). (J) Representative epifluorescence microscopic images of *gst-4::gfp* expression in L4 larvae. (K) Quantification of reporter gene expression.

See Table S6 for statistics. Error bars, SEM of at least 3 independent biological replicates. *p < 0.05; **p < 0.01; ***p < 0.001.

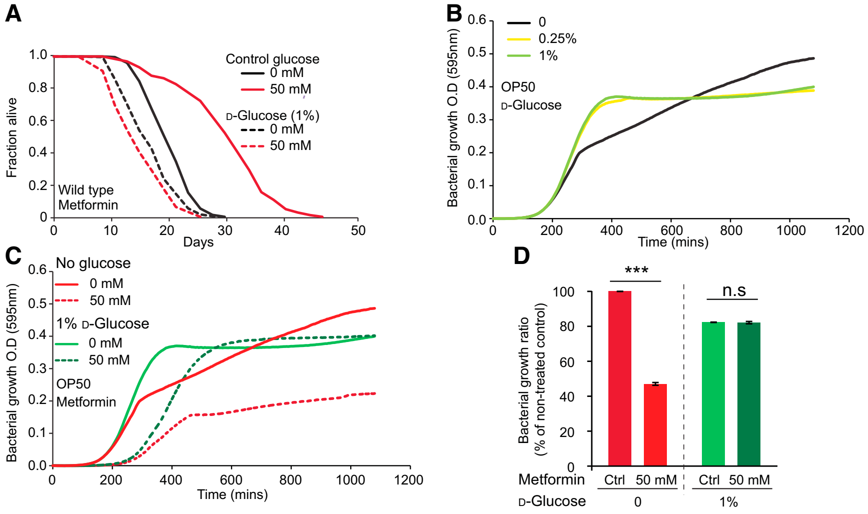

Figure S7. Glucose Supplementation Blocks Metformin-Induced Lifespan Extension, Related to Figure 7

(A) Metformin does not extend *C. elegans* lifespan in the presence of 1% D-glucose.

(B) Glucose supplementation alone slightly enhances bacterial growth.

(C and D) 1% D-glucose reverses inhibition of bacterial growth by 50 mM metformin.

See Table S7 for statistics. Error bars, SEM. *p < 0.05; **p < 0.01; ***p < 0.001.
